# Advancements in understanding the role of intestinal dysbacteriosis mediated mucosal immunity in IgA nephropathy

**DOI:** 10.1186/s12882-024-03646-3

**Published:** 2024-06-21

**Authors:** Yitao Fan, Yan Wang, Han Xiao, Hui Sun

**Affiliations:** 1 The Second Clinical Medical College of Lanzhou University, Lanzhou, 730030 Gansu China; 2 Cuiying Biomedical Research Center, Lanzhou University Second Hospital, Lanzhou, 730030 Gansu China

**Keywords:** Gut microbiome, Gut-Kidney Axis, IgA nephropathy, Gut mucosal immunity

## Abstract

IgA nephropathy, presently recognized as the foremost primary glomerular disorder, emerges as a principal contributor to renal failure globally, with its pathogenesis yet to be fully elucidated. Extensive research has highlighted the critical role of gut microbiome in the onset and progression of IgA nephropathy, underscoring its importance in accurately delineating the disease’s etiology. For example, gut microbiome dysbacteriosis can lead to the production of nephritogenic IgA1 antibodies, which form immune complexes that deposit in the kidneys, causing inflammation and damage. The gut microbiome, a source of numerous bioactive compounds, interacts with the host and plays a regulatory role in gut-immune axis modulation, earning it the moniker of the “second brain.” Recent investigations have particularly emphasized a significant correlation between IgA nephropathy and gut microbiome dysbacteriosis. This article offers a detailed overview of the pathogenic mechanisms of IgA nephropathy, specifically focusing on elucidating how alterations in the gut microbiome are associated with anomalies in the intestinal mucosal system in IgA nephropathy. Additionally, it describes the possible influence of gut microbiome on recurrent IgA nephropathy following kidney transplantation. Furthermore, it compiles potential therapeutic interventions, offering both theoretical and practical foundations for the management of IgA nephropathy. Lastly, the challenges currently faced in the therapeutic approaches to IgA nephropathy are discussed.

## Introduction

Primary Immunoglobulin A nephropathy (IgAN), commonly referred to as IgA nephropathy, stands as the globally dominant type of primary glomerulonephritis. This kidney disease was first brought to the medical community’s attention in 1968 through the pioneering work of French pathologists Jean Berger and Nicole Hinglais, subsequently named Berger’s disease. The yearly incidence rate of IgAN is documented at 2.5 cases per 100,000 individuals [1]. Epidemiological data indicate significant variations in the prevalence of IgAN based on race, gender, geographical location, and socioeconomic status. In Asian countries, the prevalence of IgAN can reach up to 50%, followed by Europe at 30%, with the lowest prevalence observed in Africa at 5% [2, 3]. Moreover, in Europe and North America, the incidence rate in male patients with IgAN is observed to be two to threefold higher compared to their female counterparts, whereas in Asia, the male to female ratio is approximately equal [4]. On average, the life expectancy of individuals with IgAN is reduced by 6–10 years [5], and 26% of patients progress to patients treated with kidney replacement therapy within ten years [6].

Currently, the confirmation of IgAN is contingent upon detecting IgA deposits within the mesangial regions of renal tissue, often accompanied by the accumulation of Complement component 3 (C3), Immunoglobulin G (IgG), or Immunoglobulin M (IgM), as observed through immunofluorescence or immunohistochemistry. Clinically, IgAN manifests through repeated occurrences of either gross or microscopic hematuria, potentially accompanied by a spectrum of proteinuria, marked hypertension, or renal insufficiency. Historically, many IgAN patients have reported exacerbations following upper respiratory or gastrointestinal infections [7], leading to a focused research interest on the impact of mucosa-associated lymphoid tissue (MALT), including the oral cavity, pharynx, and tonsils, on IgAN. However, early studies have documented correlations between IgAN and digestive system diseases, noting an increased incidence of IgAN in individuals with celiac disease and inflammatory bowel disease (IBD). This increase may be attributed to shared immunopathological mechanisms or dysregulation of the gut-kidney axis [8–12]. In recent years, with advancing research into the pathogenesis of IgAN and the gut microbiome, the theories surrounding gut-associated lymphoid tissue (GALT) and dysbacteriosis of the gut microbiome have re-emerged in the scientific discourse. Increasing evidence suggests significant correlations between IgAN and gut microbiome dysbacteriosis, thereby enriching our understanding of the etiology of IgAN and providing a foundation for the prevention, treatment, and improvement of disease prognosis in IgAN.

In this review, we delve into the epidemiology, pathological foundations, and complex pathophysiology of IgAN, with a particular emphasis on the central role of mucosal immunity in the disease process, providing researchers and clinicians with the latest insights into IgAN. We describe in detail the potential connections between IgAN and the gut microbiome, discussing how dysbacteriosis affects the recurrence of IgAN post-kidney transplantation. This section includes descriptions of the possible changes in the gut microbiome in recurrent IgAN post-transplantation and the impact of commonly used post-transplantation medications on the gut microbiome, offering new perspectives and theoretical frameworks for the mechanisms of IgAN development and recurrence post-transplantation. Furthermore, the review introduces various therapeutic approaches for IgAN, particularly those targeting the gut microbiome, and includes the latest clinical trial drugs. These insights aim to provide clinicians with strategies to improve patient outcomes. These discussions not only deepen our understanding of IgAN pathology but also provide crucial guidance for future research directions and patient management.

## Functions of the gut microbiome

The organism’s gastrointestinal tract harbors roughly 10 to the power of 14 bacterial species, alongside numerous archaea, eukaryotes, viruses, and parasites [13], constituting a complex and crucial gut microbiome ecosystem. In physiological conditions, the gut microbiome establishes a symbiotic association with the host, critically contributing to the equilibrium of gut microecology. For instance, it participates in the metabolic activities of nutrients, facilitating their absorption and utilization by the host, and maintains normal immune functions, protecting the host from pathogens in the gastrointestinal tract. However, changes in the quality and quantity of the gut microbiome, referred to as intestinal dysbacteriosis, may arise from influences including age, environment, diet, medication, and the immune system. Dysbacteriosis is associated with a plethora of diseases and states within the host, featuring diabetes [14–16], obesity [17], IBD [18–20], cancer [21, 22], cardiovascular diseases [23, 24], and kidney diseases [25], extending beyond gastrointestinal disorders. Recent studies indicate that specific alterations in the gut microbiome, particularly an increase in pathogenic bacteria and a decrease in beneficial microbes, may exacerbate mucosal immune dysregulation, thereby promoting the pathogenesis and progression of IgAN, a key pathological factor in the disease [26]. This implies that the gut microbiome possesses the capacity of modulate to systemic immune responses. Therefore, further research into the association between the gut microbiome and the host holds considerable significance for understanding the onset, progression, and outcomes of many diseases.

## The mucosal immune system and the production of IgA

Human mucosae are primarily located in the eyes, respiratory tract, gastrointestinal tract, and genitourinary tract. MALT mucosa-associated lymphoid tissue is a crucial part of the human immune system, consisting of lymphoid tissue distributed beneath various mucosal surfaces. These lymphoid tissues are made up of lymphoid nodules below the mucosal surface and microtubular mucosal cells between the epithelium. MALT includes various types, such as nasopharynx-associated lymphoid tissue (NALT), bronchus-associated lymphoid tissue (BALT), and GALT. NALT and BALT mainly consist of the tonsils, adenoids, and Waldeyer’s ring, structures that are widely present in rabbits and rats [27, 28]. In humans and mice, only BALT is present. BALT is an inducible lymphoid tissue, typically forming under conditions of infection or inflammation, and is distributed throughout the lungs, providing immune defense for the respiratory tract [28]. GALT, represented by Peyer’s patches (PPs) in the small intestine, includes isolated lymphoid follicles, crypt nodules, and scattered lymphocytes in the lamina propria. Therefore, NALT, BALT, and GALT each respond to pathogen invasion through specific immune mechanisms in their respective locations.

The gastrointestinal tract harbors a complex spatial distribution of microbiota, with significant differences observed between the microbial populations within the lumen and those on the mucosal surface [29]. The latter refers to certain microbes that are in closer proximity to intestinal epithelial cells (IECs). These cells not only receive signals and produce effector immune molecules, but also influence the function of nearby immune cells. In contrast, luminal microbes tend to have a more significant impact on energy and metabolism. The gut microbiome is indispensable for shaping intestinal immune responses. Bacterial components within this microbiota can act as antigens, stimulating both systemic and local immune responses [30]. Simultaneously, the intestinal immune system maintains tolerance to the microbiome. The gut mucosal immune system, primarily the GALT, is the body’s largest lymphoid organ [31], encompassing mesenteric lymph nodes, isolated lymphatic follicles, PPs, and lymphocytes scattered throughout the mucosa and epithelium. The synthesis and secretion of Immunoglobulin A (IgA) into the lumen characterize gut immunity. Over 80% of plasmablasts and mature plasma cells (PCs), which are activated B cells, are localized within the lamina propria (LP) of the gut mucosa. Most of these cells produce IgA, which is transported into the lumen as secretory IgA (sIgA) through IECs [32].

IgA can exist in both monomeric (monomeric IgA, mIgA) and polymeric (polymeric IgA, pIgA) forms. Humans possess two IgA subclasses: IgA1 and IgA2 [33]. In serum, IgA1 is the predominant form. On mucosal surfaces and in secretions, the ratio of IgA1 to IgA2 production varies depending on the location. IgA1 is the dominant form in secretions such as nasal mucus, tears, saliva, and milk, accounting for 70-95% of the total IgA. In contrast, IgA2 predominates in the intestinal mucosa, where it accounts for 60% of the total IgA [34]. IgA antibodies play roles in immune exclusion, neutralization, gene expression regulation, and enhanced antigen uptake [35], and their interaction with commensal bacteria maintains the homeostasis of the gut microbiome. Commensal bacteria in the gut can induce the maturation of GALT and the production of IgA. In germ-free (GF) mice, there is a significant reduction in the population of cells that produce IgA within the intestinal mucosa [36]. This reduction is likely because intestinal bacteria provide stimulatory signals that induce mucosal IgA production. Furthermore, research by Miguelangel et al. [37] found that transient intestinal colonization of GF mice with a mutant strain of *Escherichia coli* (*HA107*), which can undergo reversible transient colonization in the gut, induces an IgA response. Notably, although the traditional view holds that only live bacteria or cells can effectively stimulate the immune system, this study demonstrated that even genetically engineered bacterial strains that cannot survive without essential nutrients (such as D-alanine and meso-diaminopimelic acid) can still effectively induce an IgA response. This indicates that certain components secreted by bacteria, even when present transiently, are sufficient to elicit an immune response. However, not all commensal bacteria induce IgA production; for instance, *segmented filamentous bacteria* (*SFB*) induce IgA production in the colon of monoclonal GF mice [38, 39], while mixtures of *Clostridium* species do not. GALT identifies and modulates bacteria through sIgA produced in the intestine, thereby regulating the gut immune system [40].

There is a complex dynamic regulation between human microbiota and CD4 + T cells, both playing crucial roles in innate and adaptive immune responses [41]. Most activated CD4 + T cells are found in tissues that are continuously exposed to microbes, such as the gastrointestinal tract. In this environment, their T cell receptors trigger specific immune responses to microbial antigens under homeostatic conditions. These responses play a significant role in generating microbial-specific IgA, which is crucial for maintaining intestinal balance [42]. Thus, one of the gut immune system’s critical functions is distinguishing between commensal and pathogenic bacteria through microbial antigens, ensuring the regular maturation of the mucosal immune response. Th17 cells, representing a distinct lineage of CD4 + T cells, are characterized by their production of specific cytokines, notably IL-17 A, IL-17 F, and IL-22, significantly impacting various inflammatory responses, autoimmune diseases, transplant rejection reactions, and tumor development. Th17 cells are almost non-existent in GF mice, and changes in Th17 cells in colonized mice depend on microbial composition [43]. In identifying which microbes can induce Th17 cells, Ivanov et al. [44] found significant differences in Th17 cell numbers in the intestines of genetically identical mice from different suppliers. C57BL/6 mice from Jackson had almost no such cells, while those from Taconic had a large number of Th17 cells in the LP [45, 46]. Molecular methods to detect the microbial composition of these mice revealed the presence of *SFB* in Tac mice, later studies using feces from *SFB*-monocolonized mice confirmed *SFB* ability to induce intestinal Th17 cells [46]. Different strains of mice respond differently to immune stimuli, which can affect the reproducibility of experimental results. Additionally, the composition of the microbiota significantly influences the immune response, even among mice of the same strain but from different sources. Regulatory T cells (Tregs), a distinct subset of CD4 + T lymphocytes exhibiting immunosuppressive properties, not only balance intestinal inflammation but also help the host defend against microbial antigens by promoting specific antibody (like IgA) responses in the gut, maintaining a symbiotic interplay between the immune system and gut microbiome [47, 48]. Recent research has demonstrated that many microbes, including *Escherichia coli*, *Clostridium*, *Akkermansia*, *Fragilis*, *Streptococcus* and *Lactobacillus*, can induce Treg cells within the lamina propria of both the colon and small intestine [49, 50].

In summary, the collaborative interplay and harmonization of the gut microbiome with the mucosal immune system collectively maintain the body immune balance.

## The gut-kidney Axis in IgAN

**Fig. 1:**
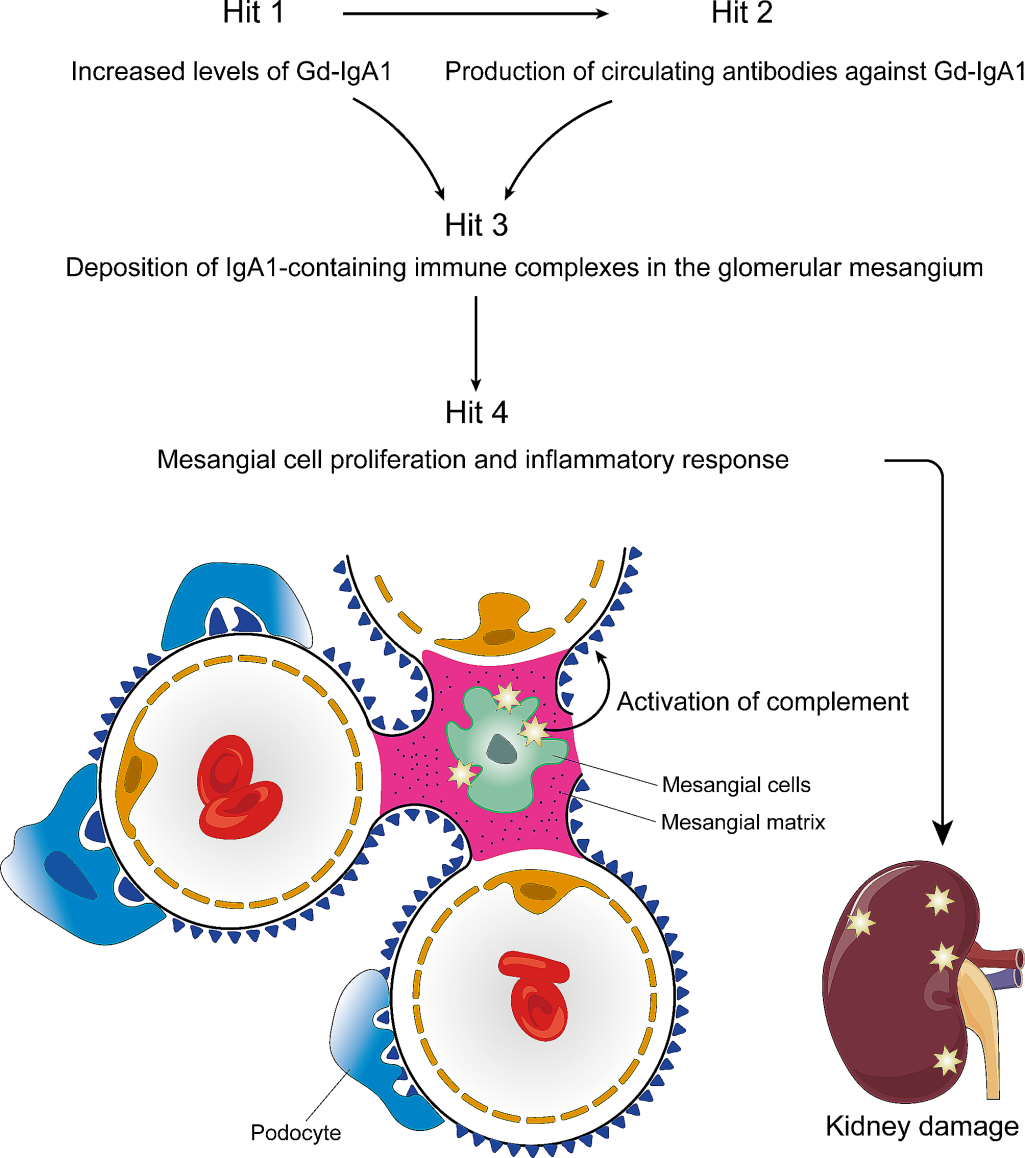
The pathogenesis of IgA nephropathy: the four-hit theory. In the pathogenesis of IgA nephropathy, the four-hit theory is a key hypothesis: (1) Hit1: Represents the production of aberrant IgA1, which may be the initiating factor of the disease process. (2) Hit2: Concurrently, IgA1 can also serve as an autoantigen, thereby inducing the production of autoantibodies. (3) Hit3: Aggregates of these aberrant IgA1 form macromolecular complexes that deposit in the kidneys. (4) Hit4: The formation and deposition of immune complexes within renal tissue trigger the proliferation of mesangial cells and the synthesis of the extracellular matrix. cytokines, and inflammatory factors, which promote inflammation and subsequently result in kidney damage

The precise pathogenesis of IgAN remains unclear to date, but the current academic consensus attributes it to the accumulation of IgA and IgA-predominant immune complexes within the mesangial regions of the kidneys, followed by secondary immune inflammatory responses. A typical example is that clinically, patients with IgA-type multiple myeloma have significantly elevated levels of polymeric IgA in circulation but rarely develop IgAN [51]. Interestingly, patients with IgAN who undergo kidney transplantation may experience recurrence of IgAN due to the redeposition of IgA and C3 in the normal transplanted kidney [52]. Conversely, if a donor kidney with IgA deposits is transplanted into a patient whose kidney failure is not due to IgAN, the mesangial IgA deposits will disappear within a few weeks [53]. According to the “four-hit” hypothesis proposed in the last decade, IgAN patients exhibit increased concentrations of galactose-deficient IgA1 (Gd-IgA1) in their circulatory system (Fig. 1). Gd-IgA1, acting as an autoantigen, induces the body to produce autoantibodies against it. Concurrently, the production of cytokines and chemokines, along with the initiation of the complement system, facilitates the aggregation of immune complexes comprising antibodies and antigens deposited in the glomerular mesangial areas [54, 55]. Due to the location, structure, and functional characteristics of mesangial cells, they are prone to the deposition of harmful residues and immune complexes [56]. In patients with IgAN, the pathogenic circulating IgA1-IgG immune complexes are relatively large (> 800 kDa), which further complicates their accumulation [56]. Furthermore, mesangial cells readily express specific receptors that facilitate the binding and accumulation of immune complexes [57]. This, consequently, stimulates mesangial cell proliferation and the production of inflammatory cytokines and matrix proteins, ultimately leading to kidney damage [58]. The source of Gd-IgA1 in circulation is still a subject of debate. Previous studies suggest that IgA1 produced by PCs within the mucosal immune system is primarily dimeric and exhibits galactose deficiency. Similarly, the IgA1 detected in immune complexes deposited in the glomeruli of IgAN patients and in their circulation is polymerized, indicative of a typical mucosal origin of sIgA1 [59]. Consequently, some scholars suggest that the Gd-IgA1 found in the circulatory system and glomeruli of IgAN patients may be derived from PCs located in the mucosa. However, it is undeniable that there is still controversy over which part of the mucosal immune system is primarily responsible for the production of Gd-IgA1.

### Controversies in tonsil mucosal immunity in IgAN

In patients with IgAN, disturbances in tonsillar mucosal immunity are considered a significant factor in its pathogenesis. This perspective has emerged and been researched over several decades since IgAN was first described. Subsequent clinical observations have shown that many IgAN patients experience worsening of their condition, with the appearance of gross hematuria, following upper respiratory tract infections such as tonsillitis. This suggests that the tonsils and upper respiratory mucosal immunity may play a role in the pathogenesis of IgAN [60–62]. This has led to increased interest in studying the role of the tonsils and their associated mucosal immunity in IgAN pathogenesis. Research on IgAN patients has shown that PCs in the tonsils can produce IgA1 and pIgA, indicating that the tonsils of some patients may be a source of increased IgA1. Furthermore, the IgA1 secreted by tonsillar lymphocytes is under-glycosylated, which may contribute to the development of IgAN [63]. Certain microbes, such as *Haemophilus parainfluenzae*, stimulate IgA synthesis in the mononuclear cells of the tonsils in IgAN patients [64]. Additionally, a study [65] found that the tonsillar microbiota of IgAN patients is similar to that of patients with recurrent tonsillitis, with a high representation of bacteria such as *Prevotella*, *Porphyromonas*, *Fusobacterium*, *Sphingomonas*, and *Treponema*, which are thought to play a role in the pathogenesis of IgAN. However, since the study did not use healthy individuals as a control group, and most IgAN patients were in the early stages of the disease, this selection may not accurately reflect the differences in tonsillar microbiota between IgAN patients and healthy individuals. Moreover, genetic diversity and environmental factors may influence the composition of microbiota and immune responses in different populations. This study focused on Japanese patients, and the small sample size may limit the generalizability of the results.

Previous scholars believed that tonsillectomy might be an effective treatment for alleviating the condition of IgAN. Unfortunately, while some patients showed a reduction in hematuria after tonsillectomy, long-term follow-up results indicated that the procedure does not prevent the progression of IgAN. The European VALIGA study [66] observed the long-term outcomes of 1147 IgAN patients and matched 41 pairs of patients who did or did not undergo tonsillectomy using propensity score matching. The results showed that tonsillectomy did not improve proteinuria or long-term renal function. Recent meta-analyses, primarily including studies from Japanese scholars, indicate that about half of the Japanese subjects showed symptom relief after tonsillectomy alone. For the remaining patients, when combined with steroid therapy, there was a trend of decreased glycosylated IgA levels [67–69]. Notably, the effectiveness of tonsillectomy in treating IgAN differs between Japanese and Caucasian patients. In Japanese patients, tonsillectomy significantly reduced proteinuria and improved renal function, possibly due to the high prevalence of tonsillar infections in this population, which may lead to different tonsillar microbiota structures. This suggests that the mechanisms of IgAN may vary between races, with Caucasians possibly requiring different treatments. Therefore, tonsillectomy for treating IgAN has not reached a global consensus and has not been widely promoted.

Overall, the role of tonsillar mucosal immunity in the pathogenesis of IgAN remains controversial. On one hand, although upper respiratory tract infections are common triggers in IgAN, the exact mechanisms by which they contribute to the disease are still not fully understood. The IgA immune response triggered by upper respiratory tract infections is complex and variable. Even though tonsillectomy has shown effectiveness in some IgAN patients, it is not universally effective for all. The efficacy of this treatment shows significant individual differences, and its long-term effects and safety are still debated. These factors make it difficult to clearly define the role of the tonsils in IgAN. On the other hand, researching the microbiota of the tonsils involves relatively complex and limited techniques, especially since collecting tonsil samples requires invasive and complicated procedures. Additionally, tonsil samples are usually small, providing limited DNA for analysis, which necessitates more precise extraction and amplification techniques. Given these challenges, researchers have had to focus on the gut mucosal immunity field for IgAN research due to the ease of sample collection, rich background data, clear systemic impact, high therapeutic potential, and technical feasibility. The Table 1 provides a detailed comparison of tonsil mucosal immunity and gut mucosal immunity in relation to IgAN.

**Table 1:** Tonsil vs. gut mucosal immunity in IgAN

Aspect	Tonsil Mucosal Immunity and IgAN	Gut Mucosal Immunity and IgAN
Similarities	(1) **Immune Mechanisms**: Both involve mucosal immune systems that capture and process pathogens, activating local T cells and B cells. (2) **IgA Production**: Both produce SIgA through the differentiation of B cells into PCs. (3) **Immune Response**: Both initiate local and systemic immune responses to prevent pathogen invasion.	(1) **Immune Mechanisms**: Both involve mucosal immune systems that capture and process pathogens, activating local T cells and B cells. (2) **IgA Production**: Both produce SIgA through the differentiation of B cells into PCs. (3) **Immune Response**: Both initiate local and systemic immune responses to prevent pathogen invasion.
Differences	(1) **Location and Function**: Tonsils are located in the oropharynx and nasopharynx, primarily dealing with inhaled pathogens. (2) **Local Impact**: IgA production in the tonsils mainly affects local immune response in the upper respiratory tract. (3) **Related Diseases**: Tonsillectomy is often used to treat chronic tonsillitis, which may impact IgAN patients.	(1) **Location and Function**: Gut mucosal immunity is located in the intestines, including GALT, primarily dealing with ingested food and pathogens. (2) **Systemic Impact**: IgA production in the gut affects both local and systemic immunity by influencing gut microbiome and systemic circulation. (3) **Related Diseases**: Gut microbiome dysbacteriosis is associated with various systemic diseases, including IgAN.
Main Theories	(1) **IgA Complex Formation**: Tonsil immune activation leads to IgA production, where IgA forms complexes with antigens that deposit in the glomeruli, contributing to IgAN. (2) **Clinical Observation**: Many IgAN patients experience symptom exacerbation following upper respiratory infections or tonsillitis, supporting the role of tonsils in IgAN. (3) **Treatment Approaches**: Tonsillectomy has shown efficacy in reducing disease severity in some IgAN patients.	(1) **Gut-Kidney Axis Hypothesis**: Gut microbiome dysbacteriosis and GALT activation lead to aberrant IgA production, which enters the bloodstream and deposits in the kidneys, affecting IgAN development. (2) **Microbiome Regulation**: A healthy gut microbiome balances immune responses and prevents excessive inflammation, protecting kidney health. (3) **Research Findings**: Multiple studies indicate a strong association between specific gut microbiome and the development of IgAN.
Controversies	(1) **Tonsillectomy**: There is controversy over the role of tonsillectomy in preventing IgAN progression; some studies show benefits, while others do not show significant effects. (2) **Mechanistic Details**: The exact mechanism by which tonsils influence IgA complex formation is not fully understood. (3) **Clinical Outcome Variability**: There is significant variability in patient responses to tonsillectomy.	(1) **Complexity of the Gut-Kidney Axis**: The exact mechanisms of the gut-kidney axis are not fully understood, and studies differ in their views on how gut microbiome influence IgAN. (2) **Individual Differences**: There is significant variability in patient responses to probiotics and dietary interventions, making clinical applications challenging. (3) **Research Methodology**: Research methods and results on the relationship between gut microbiome and IgAN are sometimes inconsistent.

The Table 1 compares the roles of tonsil mucosal immunity and gut mucosal immunity in IgAN, focusing on similarities, differences, main theories, and controversies. Both systems involve mucosal immune activation and the generation of IgA antibodies, which form immune complexes in the systemic circulation. The differences lie in their location and function: tonsils are in the throat, defending against upper respiratory infections, while the gut mucosal immune system is in the digestive tract, defending against gut infections and maintaining microbiome balance. The main theories include the formation of IgA immune complexes in the systemic circulation due to tonsil immune activation, leading to IgAN, and the gut-kidney axis hypothesis, which suggests that gut microbiome dysbiosis leads to excessive IgA antibody production and subsequent glomerular deposition. Controversies include the effectiveness of tonsillectomy in treating IgAN, with some studies supporting its efficacy and others finding no significant effect. Additionally, the complexity of the gut-kidney axis results in ongoing debate about its precise mechanisms. Overall, these comparisons and controversies highlight areas needing further exploration and validation in IgAN research.

### Gut mucosal immunity in IgAN

**Fig. 2:**
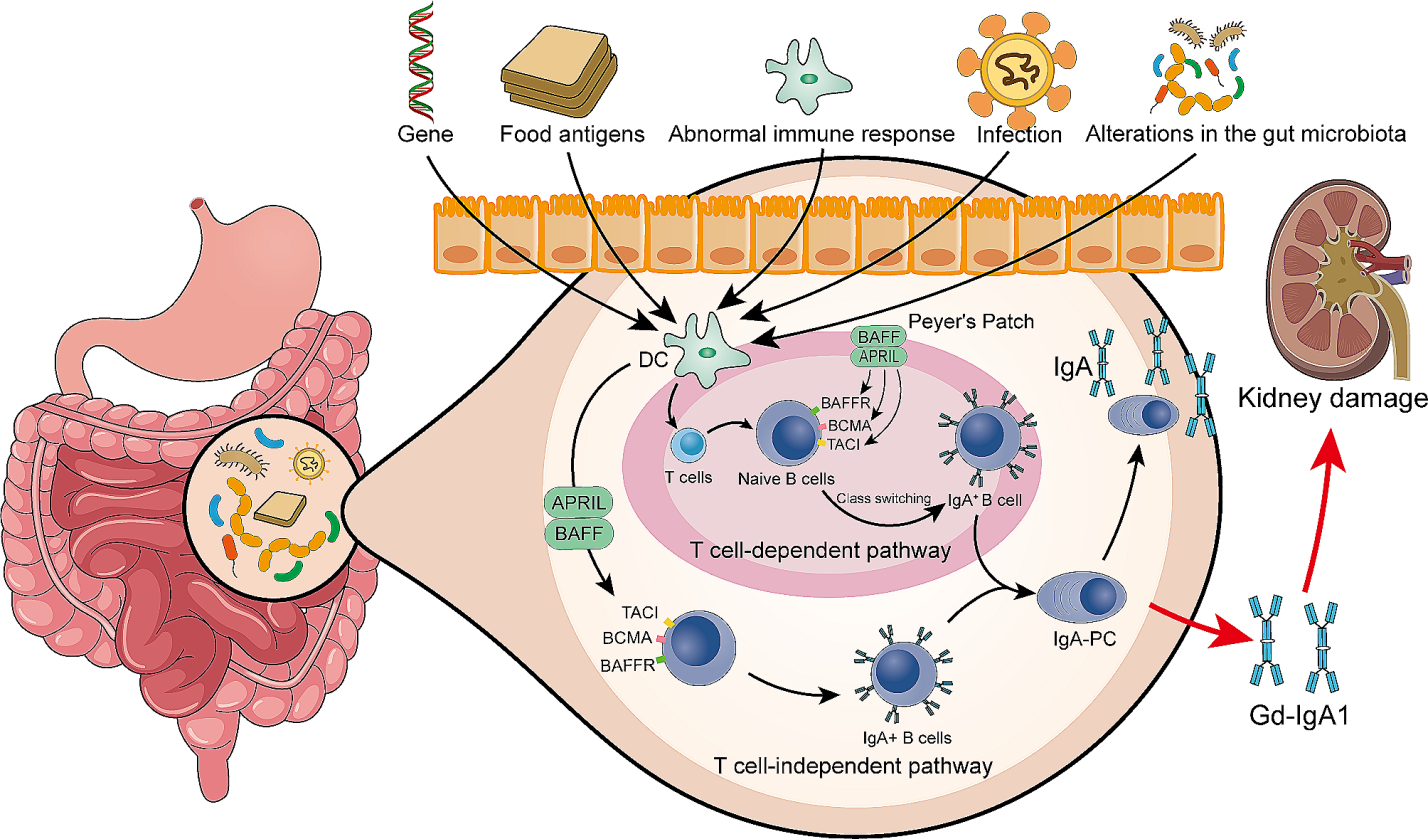
The Formation Mechanism of Gd-IgA1 in IgA nephropathy. The pathogenesis of IgAN involves a complex interplay of genetic factors, environmental triggers, and alterations in the gut microbiome. Changes in the gut microbiome impact both intestinal and systemic immune responses, possibly playing a pivotal role in the development of IgAN. The immune pathways within the gut-associated lymphoid tissue, such as PPs, include both T cell-dependent and independent routes. In the T cell-dependent pathway, dendritic cells activate T cells, which in turn assist in the activation of B cells. Under the influence of cytokines such as BAFF and APRIL, these B cells undergo class switching and ultimately differentiate into IgA-producing PCs. The T cell-independent pathway illustrates how B cells can directly receive signals through BAFF-R and TACI receptors without T cell assistance, thereby promoting the production of IgA + B cells. Crucially, the roles of TACI, BCMA, and BAFF-R receptors on B cells are vital as they enhance the cells’ survival, proliferation, and differentiation into IgA-producing cells through binding with BAFF and APRIL. This interaction of cytokines and receptors strengthens the immune response and plays a critical role in the progression of IgAN. Through these pathways, IgA + B cells evolve into PCs that migrate into the systemic circulation, producing large amounts of Gd-IgA1, a key factor in kidney damage. Gd-IgA1 forms immune complexes that deposit in the glomeruli, triggering an inflammatory response that ultimately leads to renal damage. This integrated interaction from genes to environmental factors, and to intestinal immune responses, collectively drives the pathological process of IgAN. Abbreviations: IgAN (IgA nephropathy); PPs (Peyer’s patches); BAFF (B cell activating factor); APRIL (A proliferation-inducing ligand); PCs (Plasma cells); TACI (Transmembrane activator and CAML interactor); BCMA (B-cell maturation antigen); Gd-IgA1 (Galactose-deficient IgA1);

The gastrointestinal mucosal immune system may significantly influences the development of IgAN [70]. Given that GALT is the most extensively distributed form of MALT, its relationship with pathogenic IgA is inseparable. The human gastrointestinal immune system is composed of inductive and effector sites. Inductive sites are areas where naive B cells encounter antigens. An example of these is the PPs in the small intestine, which are typical inductive sites [71]. A critical step in the generation of Gd-IgA1 entails the process of IgA class switching. Gut microbes act as antigens and are ingested by microfold cells (M cells) located within the follicle-associated epithelium. These antigens are then transferred to dendritic cells beneath the dome of PPs [72, 73]. The dendritic cells activate B cells through either T cell-dependent or independent pathways. This activation induces the transcription of the IgA constant region, driven by several cytokines, including transforming growth factor-beta (TGF-β). Ultimately, this leads to IgA class switching [74, 75]. TGF-β plays a pivotal role in stimulating B cell class switching, thereby facilitating the production of IgA. Analysis by Xiao et al. [76]. on human B cell lines has demonstrated that low concentrations of TGF-β1 (5 and 10 ng/ml) promote the production of IgA1, while high concentrations (15 and 30 ng/ml) inhibit the production of IgA1. This phenomenon may be due to the dual role of TGF-β1 in regulating B cell proliferation and activity, whereby varying concentrations of the cytokine affect cellular functions through different signaling pathways and mechanisms. Despite these differing effects of TGF-β1 concentrations on IgA1 production, TGF-β1 significantly reduces the expression of core-1 β1,3-galactosyltransferase (C1GALT1) and the molecular chaperone core-1 β3-Gal-T-specific molecular chaperone 1 (Cosmc) at all concentrations. Both C1GALT1 and Cosmc are important co-factors for IgA glycosylation linkage. Notably, in the T cell-dependent pathway, the process of B cell class switching ensues subsequent to the activation of antigen-specific T cells (Fig. 2).

In the development of IgAN, cytokines promoting IgA secretion, such as B cell activating factor (BAFF) and a proliferation-inducing ligand (APRIL), have been shown to induce abnormal mucosal immune responses to the gut microbiome due to their overactivation [77, 78]. Mucosal responses are believed to possibly be triggered by pathogenic microbes, which are identified by pattern recognition receptors such as Toll-like receptors (TLRs). These responses can promote the release of BAFF and the synthesis of IgA [79, 80]. Persistent or excessive stimulation of TLRs may lead to the generation of IgA1/Gd-IgA1 and the production of specific antibodies. McCarthy et al. [81] established transgenic mice that overexpress BAFF and demonstrated the presence of abnormally glycosylated IgA in these mice. Interestingly, male BAFF-Tg mice exhibited higher serum BAFF levels compared to female mice. After assessing BAFF mRNA levels in the spleen, liver, and kidneys using qPCR, they speculated that the sex-specific difference in BAFF expression might be due to the sexual dimorphism of the alpha-1 antitrypsin (AAT) gene cluster expressed in the kidneys. According to current research, while there is no direct inhibitory interaction between AAT and BAFF, AAT may indirectly affect BAFF expression by regulating immune cell functions, such as inhibiting monocyte and macrophage activity, and reducing the release of inflammatory mediators. Additionally, they also observed elevated serum concentrations of APRIL in IgAN patients. These findings suggest that excessive B cell survival signals may disrupt the normal balance between the gut immune system and the microbiome. This disruption highlights the possible role of the BAFF-APRIL system’s interaction with the commensal microbiome in elevating IgA levels. Evidence from human IgAN and IgAN mouse models suggests that abnormal mucosal reactions to the microbiota under the induction of cytokines like BAFF/APRIL could be a significant driver of IgAN disease. Beyond indicating that immune complexes in IgAN may originate from the gut, Gharavi et al. [82] also made significant findings through genome-wide association studies (GWAS). They identified several susceptibility loci for IgAN. These loci are primarily characterized by disruptions in the intestinal epithelial barrier and abnormal responses from gut microbes. Such abnormalities mediate the critical role of immune responses in the intestinal mucosa in the development of IgAN. In this study, they further established the genetic predisposition for IgAN in relation to climate, pathogens, and dietary factors, with local pathogens (including bacteria, viruses, spirochetes, and protozoa) showing the strongest positive correlation with the IgAN genetic risk score. Thus, the remarkable link between genetic predisposition and environmental factors may provoke alterations in the functionality of the gut mucosal immune system, predisposing to the initiation of disease.

Another strong piece of evidence supporting the connection between the intestinal mucosa and IgAN is the apparent shared genetic background between bowel diseases and IgAN. IBD is a representative disease of intestinal mucosal immune dysregulation. Patients with IBD have significantly increased IgA-producing PCs in their intestinal mucosa, leading to the local production of large amounts of IgA, elevated serum IgA levels, and a systemic IgA immune response that may affect other organs, such as the kidneys [83, 84]. This further reveals the clinical correlation between the two diseases. Currently, there is limited large-scale epidemiological data on the renal pathology of the IBD population. Ambruzs et al. [85] reported a study conducted across multiple medical centers in the United States, analyzing 33,713 kidney biopsy samples, of which 83 were from IBD patients. The results showed that IgAN was the most common glomerular disease among these IBD patients (24%, or 20 cases), with a significantly higher diagnosis frequency compared to other non-IBD kidney biopsy specimens during the same period (24% vs. 8%, *P* < 0.001), indicating a specific pathological mechanism between IBD and IgAN. The study pointed out that besides drug-induced kidney damage, systemic immune dysregulation caused by IBD itself might be an important reason for renal lesions [85]. However, as this was a retrospective study, incomplete records of patients’ medication history and disease activity might affect the accurate estimation of drug-related kidney damage incidence. A recent two-sample Mendelian randomization study [86] used single nucleotide polymorphisms as instrumental variables to analyze data from 86,640 Europeans to evaluate the causal relationship between IBD and various kidney diseases. The study found a significant causal association between IBD and IgAN, with a positive correlation with the risk of IgAN but no similar association with other kidney diseases (such as membranous nephropathy, diabetic nephropathy, etc.). This may be due to systemic immune responses caused by intestinal mucosal immune dysregulation and impaired intestinal barrier function in IBD patients, increasing the risk of IgA production and deposition. This further confirms the similarity between IBD and IgAN in terms of intestinal mucosal immune dysregulation.

The influence of certain dietary antigens cannot be overlooked. A case report from 1983 [87] on the relationship between celiac disease and IgAN revealed the potential role of gluten in causing IgAN, with patients with celiac disease showing increased circulating IgA antibodies against gliadin. Subsequently, Coppo et al. [88], through feeding BALB/c mice subjected to a diet high in gluten versus one devoid of gluten, found more IgA deposits within the mesangium in mice fed with the gluten-rich diet. CD89 (FcαRI) is a high-affinity receptor for IgA, primarily expressed on the surface of monocytes, macrophages, neutrophils, and certain dendritic cells. It regulates inflammatory responses by binding to IgA. sCD89, the soluble form of CD89, is present in the serum. Aberrantly glycosylated IgA1, such as Gd-IgA1, can bind to sCD89 to form IgA1-sCD89 complexes, which play a role in the development and progression of IgAN. Papista et al. [89], using a mouse model with humanized expression of both IgA1 and CD89 (α1KI-CD89Tg mice), found that a gluten-containing diet intensified the secretion of intestinal IgA1 and caused increased inflammation and tissue damage. Furthermore, gliadin—a minor component of gluten—and CD89 exacerbated IgAN. They did so by generating IgA1-sCD89 complexes and stimulating mucosal immune responses. However, there are significant design differences between studies. The α1KI-CD89Tg mouse model used by Papista et al., which stably expresses human IgA1 and CD89, closely mirrors the immune response and pathological features of human IgAN, providing a more accurate reflection of gluten’s impact on IgAN. In contrast, the BALB/c mice used by Coppo et al. possess natural mouse IgA, and their immune response differs from that of humans, potentially failing to fully simulate the pathological process of human IgAN. Considering that the BALB/c mouse strain was established earlier, its use in IgAN is more suitable for preliminary studies. The humanized mouse model, due to its higher pathological relevance and clinical translatability, is more appropriate for in-depth IgAN research and the evaluation of potential therapeutic approaches. Moreover, studies [90, 91] have found that in inflammatory diseases caused by autoimmunity, such as celiac disease, the intake of gluten-containing foods leads to the overexpression of zonulin. This overexpression causes a decline in tight junction proteins, which increases intestinal permeability. As a result, antigens enter the bloodstream in large quantities, possibly inducing a systemic immune response that includes the production of IgA.

However, the association between celiac disease and IgAN remains unclear. Tissue transglutaminase antibodies (tTGA) are markers of celiac disease. In a Finnish study [92] involving a cohort of 827 kidney biopsies, including 147 IgAN patients, 45 patients (5.4%) were found to have serum IgA-type anti-tTGA antibodies, of which 9 cases (1.1%) were diagnosed with celiac disease. Compared to tTGA-negative IgAN patients, tTGA-positive IgAN patients had significantly lower estimated glomerular filtration rate (eGFR) levels both at the time of biopsy and after a median follow-up of 5–6 years. Interestingly, although this study suggested an association between celiac disease and IgAN, another case-control study from New York and New Jersey refuted this association [93]. This study included 99 biopsy-confirmed IgAN patients, 96 control subjects without related diseases, and 30 diagnosed celiac disease patients. Using multivariate analysis to control for confounding variables such as age, gender, and race, and employing a broader detection range including anti-tTGA antibodies and anti-gliadin antibodies, as well as HLA-DQ2 and DQ8 genotyping associated with celiac disease, the study found no significant difference in anti-tTGA antibody levels between IgAN patients and controls. This study did not find a significant association between IgAN and celiac disease, denying the potential role of celiac disease in IgAN. The significant differences in results between these two studies might be due to variations in detection methods and demographic differences. Using more comprehensive and rigorous detection methods may better clarify the relationship between IgAN and celiac disease. Additionally, Finland and the United States have significant differences in genetic backgrounds, dietary habits, and lifestyles. In particular, the Finnish diet traditionally contains a large amount of grains, whereas American dietary culture is more diverse. These factors may significantly affect the incidence and clinical presentation of IgAN and celiac disease. Therefore, to ensure the reliability of conclusions, more standardized and large-scale studies are needed to verify these results.

In summary, these studies indicate that environmental, dietary, and genetic factors can similarly affect the gut mucosal immune system, thereby possibly participating in the pathogenesis of IgAN.

### Altered composition of the gut microbiome and IgAN

The gut microbiome has garnered increasing attention in the context of the intestinal and renal system relationship in IgAN [94]. Studies have demonstrated marked disparities in the gut microbiome composition between patients with IgAN and healthy individuals [95, 96]. Analyses of fecal specimens from individuals with IgAN versus healthy controls have shown a notable reduction in microbial diversity in IgAN patients, particularly in genera such as *Clostridium*, *Enterococcus*, *Lactobacillus*, and *Bifidobacterium*, whereas a higher proportion of genera like *Ruminococcaceae*, *Fusobacteriace*ae, *Prevotellaceae*, and *Streptococcaceae* were observed. The reduction in some probiotics, such as *Lactobacillus* and *Bifidobacterium*, may be closely related to the use of antibiotics [97] or immunosuppressants [98], as well as low-protein diets [99, 100]. Therefore, it is crucial to control for these confounding factors in research. Additionally, many inflammatory diseases can cause impaired intestinal barrier function, leading to significant changes in the gut microenvironment, which may inhibit the growth and proliferation of certain *Clostridium* strains [101]. Furthermore, Italian subjects [95] and Chinese subjects [96] differ significantly in their diets (Mediterranean diet vs. balanced mixed diet) and detection methods (pyrosequencing vs. conventional 16 S rRNA sequencing). However, both studies found a reduction in probiotics and an increase in some potential pathogens in the gut microbiome of IgAN patients, providing evidence of gut microecological imbalance in IgAN patients.

Dong et al. [102] connected the microbiota with clinical factors, proposing gut microbiome as a specific biomarker with a predictive role in the pathogenesis and diagnosis of IgAN. Specifically, the authors confirmed significant differences in the gut microbiome between IgAN patients and healthy controls, as well as between IgAN patients and those with membranous nephropathy. Levels of *Escherichia* and *Shigella* were notably higher in IgAN patients in comparison to the control group, while levels of *Rothia* and *Haemophilus* were lower, though further investigation is warranted to establish a causal association between gut microbiome and IgAN. *Escherichia* and *Shigella* are both members of the phylum *Proteobacteria* and have the ability to grow and reproduce rapidly. They can competitively consume specific nutrients, such as galactitol, thereby inhibiting the growth of other bacteria [103]. These bacteria exhibit strong adaptability to environmental changes and can evade host immune responses through various mechanisms, further consolidating their ecological niche. Additionally, they are capable of forming biofilms, which increase their adhesion and antibiotic resistance, giving them a competitive edge over other gut microorganisms [104]. However, this competition can disrupt the balance of the gut microbiome, leading to an overgrowth of *Proteobacteria* and a reduction in beneficial bacteria such as *Bifidobacterium* and *Lactobacillus* [105]. In a fascinating observation, the abundance of *Escherichia*-*Shigella* was notably elevated in patients with IgAN, displaying a significant expansion advantage within the gut microbiome [96, 102, 106]. Zhao et al. [107] identified that in untreated IgAN patients, the proliferation of the *Escherichia*-*Shigella* genus was associated with a reduction in the intestinal microbial diversity inherent to these individuals. Furthermore, a substantial reduction in this bacterial group following immunosuppressive treatment correlated with clinical remission. However, these findings have thus far been investigated solely within the Chinese population, and future studies are warranted across a broader range of ethnic groups to verify these results. Tang et al. [108] observed significant differences in the bacterial composition at the genus and phylum levels between IgAN patients and healthy individuals. The gut microbiome of IgAN patients exhibited lower α-diversity compared to the healthy group, indicating a reduced richness and evenness of microbial species. They also discovered that the levels of Gd-IgA1 in urine have a higher diagnostic value than those in serum. In clinical practice, the levels of Gd-IgA1 in urine can reflect glomerular damage and inflammation. Compared to serum IgA1 levels, urinary Gd-IgA1 more directly reflects renal pathology. Urine sample collection is convenient and non-invasive, making it suitable for large-scale screening and monitoring. However, the diagnosis of IgAN in clinical practice still primarily relies on renal biopsy, while the detection of Gd-IgA1 in urine has not yet been widely adopted for clinical diagnosis. Moreover, specific genera such as *Coprococcus*, *Dorea*, *Bifidobacterium*, *Blautia*, and *Lactococcus* showed a strong inverse correlation with urine Gd-IgA1 levels [108]. This suggests that these bacteria may play a protective role in the pathophysiology of IgAN.

*Actinobacteria* is a major phylum of Gram-positive bacteria known for their diversity and metabolic capabilities, which are crucial for maintaining gut microbial homeostasis. In other renal diseases, such as chronic kidney disease (CKD), an increased abundance of *Actinobacteria* can be observed in patients [109]. Gut microbiome metabolites, such as indoxyl sulfate and p-cresyl sulfate, are considered important factors in the deterioration of renal function in CKD patients [109, 110]. A Mendelian randomization analysis also unveiled the pivotal role of the gut microbiome in the pathogenesis of IgAN [111]. It specifically highlighted a strong causal association between the presence of *Actinobacteria* and an increased risk of IgAN. It is reasonable to speculate that a high abundance of *Actinobacteria* may induce or exacerbate inflammatory responses in the gut and kidneys through its metabolites, thereby promoting the pathological progression of IgAN. Although the Bonferroni correction test did not show a significant effect, the analysis demonstrated that β-hydroxybutyrate is correlated with a reduced risk of IgAN, suggesting a potential therapeutic target for intervention [111].

Additionally, Gleeson et al. [112] provided evidence regarding the role of *Akkermansia muciniphila* in the pathogenesis of IgAN, further emphasizing the importance of the gut-kidney axis in renal diseases. Mucolytic bacteria, such as *A. muciniphila*, are gut bacteria capable of degrading mucin, which can modify IgA1 by removing its glycosylation, thereby creating new antigenic epitopes. These newly formed epitopes are recognized by autoantibodies, which are associated with the formation of immune complexes that deposit in the kidneys, thereby possibly inducing or exacerbating IgAN. Therefore, variations in the abundance of specific bacteria like *A. muciniphila* possibly correlate with the severity of the disease. This phenomenon was validated in α1KI-CD89Tg mice. In the gut of these mice, deglycosylated IgA1 by *A. muciniphila* can cross the intestinal epithelial cells into the bloodstream and eventually deposit in the glomeruli, exacerbating the IgAN phenotype. Notably, this study specifically examined the correlation between alpha-defensins and the abundance of *A. muciniphila* [112]. Alpha-defensins are small antimicrobial peptides with broad-spectrum antimicrobial activity that regulate host immune responses and maintain gut microbiome balance and intestinal barrier function [113]. It can be reasonably speculated that in IgAN patients, impairment of the intestinal barrier may trigger the production of inflammatory mediators. These mediators can further disrupt the redox gradient of the gut, cause dysbacteriosis, and lead to changes in pH levels. Moreover, metabolic products secreted by *A. muciniphila* may exacerbate these processes. These factors together may cause alpha-defensins, particularly DEFA6, to lose their effective inhibition of *A. muciniphila*, resulting in the overgrowth of *A. muciniphila* in the gut, thereby exacerbating the pathological progression of IgAN. Therefore, given the importance of *A. muciniphila*, its mechanisms of action in IgAN patients warrant further in-depth research.

Lipopolysaccharide (LPS), a constituent of the outer membrane of Gram-negative bacteria, has been found to exert its effects through Toll-like receptor 4 (TLR4) located in the cell membranes of target cells, suggesting that LPS might inhibit Cosmc mRNA expression by stimulating TLR4 activation in peripheral B cells, leading to the excessive production of polymeric Gd-IgA1 [114]. Additionally, changes in the intestinal barrier and heightened gut permeability in individuals with IgAN promote the absorption of LPS into the bloodstream, exacerbating the progression of the disease [114].

Studies conducted by Tang et al. [108] and Zhu et al. [115] further indicates that dysbacteriosis of the gut microbiome in patients with IgAN triggers the activation of TLR4. This activation not only increases the production of Gd-IgA1 but also stimulates the TLR4/MyD88/NF-κB signaling pathway, thereby promoting the production of inflammatory cytokines and the enhanced expression of BAFF/APRIL. Notably, these two studies have certain conflicts in their interpretation of gut-immune interactions in IgAN. Both studies excluded the effects of common medications, probiotics, and other diseases. The study by Tang et al. [108] primarily involved 25 newly diagnosed and untreated IgAN patients, focusing on urine samples. They confirmed that LPS could inhibit Cosmc mRNA expression through TLR4 activation, leading to increased production of Gd-IgA1. They emphasized that increased gut permeability in IgAN patients facilitates the absorption of LPS into the bloodstream, exacerbating disease progression. In contrast, the study by Zhu et al. [115] included 48 untreated IgAN patients and 22 treated IgAN patients. By analyzing serum and fecal samples, it was found that untreated IgAN patients exhibited more pronounced gut dysbacteriosis, particularly an increase in Escherichia-Shigella. Although symptoms were alleviated in treated patients, they still exhibited characteristics of gut dysbacteriosis and intestinal barrier damage, such as elevated levels of proinflammatory cytokines and BAFF/APRIL. These abnormalities remained significant when compared to healthy controls. Zhu et al. emphasized that gut dysbacteriosis (specific bacterial changes) is the main factor leading to TLR4 activation and IgAN pathogenesis. They proposed a broader mechanism, including changes in gut microbiome composition and its direct impact on TLR4 signaling. Additionally, they demonstrated the overall impact of gut microbiome from untreated and treated IgAN patients by transplanting it into antibiotic-treated mice through fecal microbiota transplantation (FMT). Even the gut microbiome from treated patients still induced kidney damage in mice, providing a comprehensive perspective on gut-immune interactions. Therefore, both studies highlighted that LPS-induced TLR4 activation leads to decreased Cosmc mRNA expression and increased Gd-IgA1 production, but these different perspectives lead to varying interpretations of the underlying mechanisms and therapeutic targets. These findings provide valuable references for future research.

The pathogenesis of IgAN is complex and multifactorial. Mucosal immune responses in the tonsils and intestines, along with associated gut microbiome, play significant roles in the development of IgAN. The tonsils act as immune barriers in the upper respiratory tract, with immune cells such as B cells and T cells being activated upon antigen exposure and subsequently secreting IgA. During upper respiratory tract infections, immune responses triggered by pathogens may lead to the excessive production and abnormal glycosylation of IgA in the tonsils, forming pathogenic IgA complexes that cannot be cleared normally. These abnormal IgA complexes can reach the kidneys via the circulatory system and deposit in the mesangial regions of the glomeruli, causing glomerular damage. The balance of gut mucosal immunity and gut microbiome also plays a crucial role in the pathogenesis of IgAN. The gut is the largest immune organ in the body, and maintaining the balance of gut microbiome is essential for immune homeostasis. Dysbacteriosis, characterized by an increase in pathogenic bacteria and a decrease in beneficial bacteria, may affect the gut-kidney axis’s immune regulation, leading to systemic immune abnormalities. This immune dysregulation may promote abnormal glycosylation and metabolism of IgA, thereby worsening the condition. Additionally, metabolites produced by the gut microbiome may affect renal health through the gut-kidney axis, exacerbating the progression of IgAN. In addition to these primary factors, genetic background, genetic regulatory abnormalities, and aberrant activity of glycosyltransferases may also contribute to the pathogenesis of IgAN. The main factors related to the pathogenesis of IgAN are presented in Table 2. Despite the importance of these factors, the abnormalities in tonsillar and gut mucosal immunity and their impact on IgA glycosylation and metabolism are undoubtedly central to the pathogenesis of IgAN. In summary, the pathogenesis of IgAN involves complex immune regulation and microbial dysbacteriosis, with mucosal immune abnormalities and gut microbiome imbalance playing critical roles.

**Table 2:** Factors associated with the pathogenesis of IgAN

Factor	Alteration	Significance
Gut Microbiome Dysbacteriosis	Altered composition of gut microbiome	Impaired mucosal immunity and increased IgA production
Cytokines	Elevated levels of TNF-α, IL-6, IL-8	Promotes inflammation and glomerular injury
Genetics	Mutations in genes related to immune response	Genetic predisposition to IgAN
Diet	High intake of gluten and dairy	Potentially increases mucosal immune response
Mucosal Immunity	Abnormal IgA1 glycosylation	Leads to formation of immune complexes
Complement System	Increased activation of alternative pathway	Enhances glomerular inflammation and damage
Infections	Recurrent upper respiratory infections	Triggers abnormal IgA1 production
Oxidative Stress	Increased oxidative stress markers	Contributes to kidney damage and progression of IgAN
Mesangial Cells	Overexpression of TLRs	Induces inflammatory responses and immune complex deposition
Epigenetics	Altered DNA methylation patterns	Regulates gene expression involved in immune response

IgAN is a complex immune-mediated glomerular disease influenced by various biological processes. Firstly, gut microbiome dysbacteriosis, involving changes in microbiota composition and diversity, impairs gut barrier function, leading to increased antigen translocation and immune responses. Elevated levels of pro-inflammatory cytokines (e.g., TNF-α, IL-6, IL-8) promote inflammation and glomerular cell proliferation, resulting in matrix expansion and fibrosis, thus accelerating IgAN progression. Genetic mutations in immune-related genes (e.g., HLA region, complement system genes, glycosylation enzymes) increase IgAN susceptibility. Dietary factors, such as high intake of gluten and dairy, may alter gut microbiome and promote immune responses. Abnormal glycosylation of IgA1 leads to Gd-IgA1, which forms immune complexes that deposit in the glomeruli, causing inflammation and damage. Overactivation of the complement system exacerbates glomerular inflammation, leading to cell injury and tissue fibrosis. Recurrent upper respiratory tract infections, particularly streptococcal infections, trigger immune responses that result in abnormal IgA1 production and immune complex formation. Oxidative stress induces inflammation and apoptosis, accelerating glomerular damage. Overexpression of Toll-like receptors on mesangial cells triggers immune responses, promoting cell proliferation and matrix accumulation. Epigenetic changes affect gene expression, regulating immune-related genes and altering susceptibility to IgAN and disease progression. Understanding these factors and mechanisms is crucial for developing effective therapeutic approaches.

## The current status of recurrent IgAN post-kidney transplantation

The global annual incidence of IgAN in adults is estimated at 2.5 per 100,000, with approximately 26% of patients requiring kidney transplantation within ten years of a confirmed diagnosis [1, 6]. However, one of the most common causes of graft loss ten years post-transplantation is the recurrence of the primary disease [116]. Thus, the recurrent IgAN post-kidney transplantation presents a significant challenge. The incidence of recurrent IgAN post-kidney transplantation varies by location, with studies reporting rates ranging from 30 to 60%, depending on local biopsy methods and follow-up strategies [117–119]. While previous studies considered recurrent IgAN to manifest as a benign clinical course, recent research indicates that recurrent IgAN may lead to impaired graft function or even loss in some patients [117, 120].

### Gut microbiome’s role in IgAN post-kidney transplantation

The composition and diversity imbalances of the gut microbiome play a crucial role in the prognosis of kidney transplant recipients, significantly impacting the host’s immune system and the production of inflammatory cytokines [121–125]. The success of kidney transplantation largely depends on the recipient’s degree of immune response to the donor kidney. Post-operatively, transplant recipients not only undergo immunosuppressive therapy but also receive various antimicrobial drugs for prophylactic anti-infection treatment in the early stages. These drugs can alter the composition of the gut microbiome and even cause dysbacteriosis, leading to damage to the intestinal mucosal barrier. This damage allows pathogenic bacteria to enter the bloodstream, causing infections and the recurrence of the primary disease, among other complications [123, 126]. This has been validated both in animal transplant models and in patients. Wu and his team [127] used BALB/c (H2-Kd genotype) mice as donors and C57BL/6 (H2-Kb genotype) mice as recipients for kidney transplantation. The H-2 complex (Histocompatibility-2) differences between these two strains effectively simulated the immune rejection seen in allotransplantation. Without pre-treatment with immunosuppressants and antibiotics, mice on a normal diet post-transplant showed a decrease in gut microbiome α-diversity and an increase in the relative abundance of *A. muciniphila* from *Verrucomicrobia*, a phenomenon not observed in the control group. The role of *A. muciniphila* in this study is similar to the findings of Gleeson et al. [112], suggesting its increase post-transplantation may be related to immune response and changes in the gut mucus layer. Notably, a study has demonstrated that antibiotic pretreatment can delay the rejection of transplanted organs in liver transplant models [128]. This effect may be attributed to the liver’s role as a metabolic and detoxifying organ, making it sensitive to inflammation and cellular damage. Antibiotics can alleviate ischemia-reperfusion injury and post-transplant hepatocyte death by modulating specific molecular and cellular mechanisms, thereby enhancing transplant success rates. However, there exists a complex interaction between the kidneys and the gut. Antibiotic pretreatment may reduce the diversity and abundance of beneficial gut microbiome, disrupting the gut microbiome. This disruption possibly leads to adverse immune responses and accelerate rejection. Furthermore, Wu et al. emphasized the important role of a high-fiber diet in enhancing the diversity of the gut microbiome in mice after kidney transplantation. Compared to a normal diet, mice on a high-fiber diet post-transplant produced more short-chain fatty acids, which may be related to the increased relative abundance of certain gut microbiome, including *Bifidobacterium* and *Bacteroides* [127]. These changes help to mitigate the immune rejection post-transplantation and prolong the survival time of the graft.

This finding is also applicable to the gut microbiome of patients who have undergone kidney transplantation. After amplifying the 16 S rRNA genes V3-V4 hypervariable regions using PCR and sequencing with MiSeq Illumina technology, Fricke et al. [129] found an increase in the abundance of *Firmicutes*, *Proteobacteria*, and *Actinobacteria* in patients one month and six months post-transplant. Subsequently, Zaza’s study also confirmed this, showing that the increase in the *Firmicutes*, *Proteobacteria*, and *Actinobacteria* in post-transplant patients was approximately the same [130]. Sampaio discovered in a 2023 cross-sectional study significant changes in the gut microbiome of kidney transplant recipients before and three months after transplantation, with a decrease in diversity and an increase in the relative abundance of members of the *Proteobacteria*, particularly the family *Enterobacteriaceae* [131]. Notably, an increase in the *Firmicutes*, *Proteobacteria*, and *Actinobacteria* is also observed in the intestines of patients with IgAN [132–135], especially with *Actinobacteria* showing significantly higher relative abundance compared to controls, consistent with previous studies [111, 134]. These studies also show that the abundance of *Firmicutes* and *Bacteroidetes* changes after kidney transplantation. The ratio of *Firmicutes* to *Bacteroidetes* plays an important role in maintaining gut microbiome balance. Some members of one phylum help to maintain the normal quantity of potentially pathogenic bacteria from the other phylum [136]. Therefore, changes in the abundance of *Firmicutes* and *Bacteroidetes* may potentially affect the patient’s physiological condition. Additionally, Fricke et al. found that changes were more significant in the early post-transplant period (one month), while some microbiota began to recover or further change in the later period (six months). These observed changes in the microbiota may be associated with the use of proton pump inhibitors (PPIs), cyclosporine A (CsA), and mycophenolate mofetil (MMF), reflecting the significant impact of kidney transplantation surgery and subsequent immunosuppressive therapy on the gut microbiome. Overall, these studies underscore the importance of the gut microbiome in the health of kidney transplant recipients and suggest that changes in the gut microbiome can be used to predict adverse outcomes post-transplantation, such as graft rejection, infections, and even recurrence of the primary disease.

### Metabolism of recurrent IgAN post-kidney transplantation therapeutic drugs by the gut microbiome

For patients with IgAN experiencing severe loss of kidney function, transplantation is often the best option. However, effective treatment methods for recurrent IgAN post-transplantation are currently lacking. Current treatment strategies primarily include steroids, calcineurin inhibitors, other immunosuppressive drugs, and plasma exchange, yet these approaches have not demonstrated significant efficacy. It is evident that there is a bidirectional interaction between the gut microbiome and immunosuppressants, where gut microbes can affect the absorption and metabolism of immunosuppressive agents. Concurrently, the use of immunosuppressants may also lead to changes in the composition of the gut microbiome. Zimmerman et al. [137] have shown that a significant number of gut bacteria can substantially metabolize various drugs, revealing how individual variations in the microbiome could lead to differences in drug metabolism, which has important implications for clinical efficacy and toxicity.

Immunosuppressants are the primary treatment for primary IgAN, yet no immunosuppressant has effectively prevented the histological recurrence of the disease. MMF, an anti-proliferative immunosuppressant, acts on both T and B lymphocytes, thereby reducing the deposition of IgA products in patients with IgAN. Early pharmacokinetic studies found that the active metabolite of MMF, mycophenolic acid (MPA), is converted in the liver to mycophenolic acid glucuronide (MPAG) and then excreted into the intestines via the bile. Bacterial β-glucuronidase (GUS) in the intestines can convert MPAG back to MPA, allowing for its reabsorption into the bloodstream. This enterohepatic recirculation process can be influenced by the composition of the gut microbiome [138]. Given that MMF can cause various gastrointestinal adverse effects, including diarrhea, its pharmacologic effects are likely closely related to the gut microbiome. Flannigan et al. [139] found that mice fed with MMF-containing diet exhibited significant weight loss and notable colonic inflammation. Changes in the gut microbiome composition detected via 16 S rRNA Illumina sequencing indicated an increase in the *Proteobacteria* (particularly *Escherichia* and *Shigella*) and enrichment of genes associated with LPS biosynthesis in these mice. However, these effects were not observed in GF mice, suggesting that a complete gut microbiome is a necessary condition for the gastrointestinal toxicity induced by MMF. Taylor et al. [140] discovered that the use of vancomycin reduced the activity of intestinal GUS in mice, reversing the weight loss and other gastrointestinal adverse effects caused by MMF. This suggests a link between the increase of *Clostridium* and *Bacteroides* in the mouse gut, the GUS activity they produce, and MMF-induced gastrointestinal adverse effects. Notably, Taylor et al. primarily used vancomycin as a single antibiotic during the experiment, differing from the antibiotic selection in the study by Flannigan et al. Vancomycin primarily inhibits Gram-positive bacteria by binding to the peptidoglycan layer of the bacterial cell wall, which is exposed in Gram-positive bacteria but protected by an outer membrane in Gram-negative bacteria, rendering it ineffective against Gram-negative bacteria [141]. The choice of vancomycin as a single antibiotic in the study of GUS enzyme activity was likely to reduce unnecessary variable interference. However, it should be noted that vancomycin effectively eliminates the increase in the abundance of *Bacteroidia* (mostly Gram-negative bacteria) caused by MMF treatment, and many of these bacteria are GUS producers, which seems contradictory to the mechanism of vancomycin [140, 141]. Therefore, it is reasonable to speculate that although vancomycin does not have direct activity against Gram-negative bacteria, it may indirectly affect the gut microbiome. Taylor et al. used vancomycin to target certain Gram-positive bacteria in the gut, potentially leading to overall changes in the gut microbiome. These changes might indirectly affect Gram-negative bacterial populations by altering the competitive dynamics within the gut microbiome. In the study by Taylor et al., in addition to feeding mice with MMF-containing diet to simulate long-term drug exposure, they also administered solutions of GA and MPA via rectal injection [140]. This approach allowed researchers to observe the local effects of the drugs more directly without the interference of systemic factors. Consequently, these differences not only affected the distribution and metabolism of MMF in the body but also potentially led to different toxicity profiles and changes in the microbiota, thereby impacting the validity and comparability of the study’s conclusions. Simpson and colleagues conducted a study using metagenomics and activity-based protein profiling to analyze the microbiota in the feces of kidney transplant recipients [142]. They found that certain intestinal bacterial enzymes, particularly flavin mononucleotide (FMN)-binding GUS, play a significant role in the reactivation of the drug MPA within the gastrointestinal tract. This reactivation is associated with increased gastrointestinal toxicity observed in patients treated with the immunosuppressant MMF. It is particularly noteworthy that the results from metagenomic data did not align with those from proteomic data in this study. Metagenomic data failed to show a correlation between MPA reactivation rates and either the microbial composition or the presence of specific GUS genes. However, proteomic data revealed a significant correlation between FMN-binding GUS enzymes and the rate of drug reactivation. This discrepancy is likely because metagenomics only indicates the presence of genes, which does not necessarily reflect whether these genes are transcribed and translated into functional proteins. In contrast, proteomics directly measures the actual expression levels and functions of proteins, thus compensating for the limitations of metagenomics. Additionally, the study compared fecal samples from kidney transplant recipients with those from healthy individuals, but the small sample size and inconsistent timing of sample collection from the transplant recipients might have affected the reliability of the results. Geographic differences (New York vs. North Carolina) could also have contributed to the variations in the microbiota [142]. Overall, the presence and activity of these specific intestinal bacterial enzymes are crucial for understanding the adverse effects experienced by patients, highlighting the important interactions between drug metabolism and the gut microbiome.

Tacrolimus, a macrolide antibiotic-type calcineurin inhibitor, has immunosuppressive effects that are 10 to 100 times stronger than CsA, both in vivo and in vitro, making it a first-line medication for anti-rejection therapy and treating recurrent IgAN post-kidney transplantation. A cross-sectional study has shown that the pharmacokinetics of tacrolimus are closely linked to the variety of the gut microbiome in patients with kidney transplants [143]. This implies that although previous research suggested genetic polymorphisms (such as gene mutations) influence the pharmacokinetics parameters of tacrolimus to some extent [144], it is now recognized that the composition and diversity of the gut microbiome are also significant factors. This may explain why there is substantial variability in the pharmacokinetics of tacrolimus among different individuals. Changes in the gut microbiome induced by tacrolimus treatment may lead to adverse effects, such as hypertension, diabetes, and diarrhea. In animal models, intraperitoneal injection of tacrolimus in mice led to a decrease in microbial diversity, an increased *Firmicutes*/*Bacteroidetes* ratio, and a reduction in bacteria producing acetate and butyrate [145]. However, the gut microbiome’s response to tacrolimus may vary with different dosages of the drug. Jiang et al. [146] found in a rat liver transplant model that a moderate dose of tacrolimus (0.5 mg/kg) maximally preserved the structure and function of the transplanted liver, and enhanced the diversity of the gut microbiome as well as the abundance of beneficial bacteria such as *Bifidobacterium* and *Faecalibacterium prausnitzii*. In contrast, higher (1 mg/kg) and lower doses (0.1 mg/kg) of tacrolimus led to varying degrees of rejection and a significant reduction in *Bifidobacterium* and *F. prausnitzii*. This study was conducted solely in a liver transplant model, and given the liver’s unique immune environment and rich blood supply, its response to immunosuppressants and metabolism may differ from other organs. Additionally, the specific interactions of the gut-liver axis post-liver transplant might not be fully applicable to other organs. This indicates that when conducting specialized organ studies, the unique immune characteristics, drug metabolism, and interactions with the gut microbiome of each organ need to be considered. In clinical trials, there are also comparative studies on different doses of tacrolimus. A retrospective study [147] comparing different initial doses in 127 kidney transplant recipients over one year found that the higher dose group (0.075 mg/kg) was more likely to experience supratherapeutic levels compared to the lower dose group (0.05 mg/kg). Although the lower dose group achieved the target therapeutic level (6–10 µg/L) within 14 days post-transplant at a higher proportion, this did not translate into better clinical outcomes, suggesting that early tacrolimus level variations may not significantly affect long-term results in kidney transplant recipients. Another prospective randomized controlled non-inferiority trial [148], which included 398 kidney transplant recipients with a follow-up period of 6 months, compared the effects of a fixed low dose (5 mg/day) and concentration-controlled standard dose of tacrolimus. The study found no significant differences in efficacy and safety between the fixed low dose and standard dose groups at 6 months post-transplant, indicating that the low dose regimen was not inferior in maintaining immunosuppression. Therefore, compared to standard or higher doses of tacrolimus, a low dose regimen may be more feasible in clinical practice. Additionally, a 2024 study by Li et al. [149] found that vancomycin effectively alleviated hyperglycemia induced by tacrolimus in mice by inhibiting the activity of GUS in the gut bacteria. This inhibition reduced the hydrolysis of bile acid-glucuronide conjugates, altered bile acid metabolism, and enhanced the secretion of glucagon-like peptide-1, thereby helping to regulate blood sugar levels. In terms of methods for detecting bacterial metabolites and enzyme activity, the studies by Taylor et al. [140] and Li et al. [149] differ. Taylor et al.‘s study mainly relied on known genomic databases to predict the proportional abundance of GUS genes, rather than directly measuring the actual enzyme activity. In contrast, Li et al. used an improved chromatographic method to quantitatively detect GUS activity in feces, thus providing more accurate enzyme activity data. Although both studies employed in vivo imaging methods, Taylor et al. also included detailed in vitro biochemical assays, which helped to validate the accuracy of the in vivo observations. Overall, both studies indicate that vancomycin or other GUS inhibitors could serve as potential clinical interventions to manage drug-related toxic reactions from immunosuppressants. In summary, tacrolimus influences the host by altering the composition of the gut microbiome, particularly in terms of immunosuppression and metabolism. Additionally, the gut microbiome’s response to tacrolimus exhibits dose-dependency, suggesting that the gut microbiome may play a role in the metabolic process of tacrolimus, thereby affecting the variability in drug responses among different patients with recurrent IgAN post-kidney transplantation.

## Treatments

### Antibiotics

The influence of antibiotics on the gut microbiome is manifest. A study by Chemouny et al. [30], utilizing a humanized mouse model of IgAN, showed that the administration of broad-spectrum antibiotics such as metronidazole, vancomycin, and ampicillin effectively cleared IgA1 deposits from the glomerular mesangium and reduced proteinuria. Additionally, these antibiotics disrupted the formation of hIgA1-mIgG immune complexes in the circulation. Likewise, Di Leo et al. [150] assessed the therapeutic efficacy of rifaximin in IgAN. Rifaximin is a non-absorbable oral antibiotic that primarily inhibits bacterial RNA synthesis by binding to the β-subunit of bacterial DNA-dependent RNA polymerase. It exhibits bactericidal and bacteriostatic activity against both Gram-positive and Gram-negative bacteria. The study results indicated that rifaximin reduced levels of IgA1-sCD89, creatinine and mouse IgG-IgA1 complexes, as well as the deposition of IgA1 in the glomeruli. Notably, unlike the broad-spectrum antibiotics used in Chemouny et al.‘s study, which may affect the gut and systemic microbiota in mice, rifaximin primarily acts in the gut [151]. This may result in different therapeutic mechanisms and outcomes. Despite its demonstrated potential in animal models, the short duration of existing studies means that the long-term efficacy and safety of rifaximin remain unproven. Further research is needed to evaluate its adverse effects and long-term use risks to ensure treatment adherence and safety in IgAN patients. In other gastrointestinal diseases, rifaximin can modulate gut inflammation and immune responses by activating the pregnane X receptor, thereby protecting intestinal barrier function [152, 153].

However, due to the potential adverse consequences of antibiotic misuse, such as *Clostridioides difficile* infection and antibiotic resistance, antibiotics should be used with caution [80]. In this context, selectively removing individual species or strains of bacteria from the microbial community may be more desirable than broad-spectrum antibiotics. Ting et al. [154] have developed programmable inhibitory cells (PICs), which guide the Type VI secretion system to exhibit effective antimicrobial activity against specific target bacteria. The benefit of this approach lies in using PICs to efficiently clear target bacteria through specific bacterial antigens, while having minimal impact on non-target bacteria. This demonstrates a selective clearance effect on low-abundance target bacteria within complex microbial communities. By preserving the normal microbial community’s composition, this approach enhances safety and stability. Therefore, PICs could evolve as a viable alternative to conventional antimicrobial agents. In the future, IgAN treatment may increasingly rely on precise microbial modulation strategies rather than broad-spectrum antibiotics.

### Probiotics

Probiotics refer to a category of live microorganisms that inhabit the human body and confer benefits to the host [155]. Probiotics modulate the balance of gut microbiome, promote nutrient absorption, and preserve gastrointestinal wellbeing, thereby effectively preventing gastrointestinal infections and inflammation [156]. It has been reported that in CKD, probiotics can enhance gut barrier function, reducing the production of uremic toxins, blood urea nitrogen, and some inflammatory markers [157]. Building on this, Soylu et al. [158] conducted an assessment of the impact of *Saccharomyces boulardii* on experimental IgAN in murine models. The experiment included four groups of BALB/c mice: one group received oral poliovirus vaccine (OPV) alone, the second group received OPV and *S. boulardii*, the third group received only *S. boulardii*, and the fourth group served as the control. Renal tissue pathology was evaluated using optical microscopy, immunofluorescence, and electron microscopy. The results showed that group 1 mice exhibited significant IgA deposition and mesangial proliferation, while groups 2, 3, and 4 did not show significant pathological changes. They confirmed that *S. boulardii* could reduce the production of systemic IgA, successfully preventing mouse IgAN induced by oral poliovirus vaccine. Given that live and heat-killed bacteria exhibit different effects in various models, live bacteria may offer superior benefits in certain treatments, although heat-killed bacteria can also be beneficial in specific contexts [159]. The observed effects in this study were attributed to the biological activity of live *S. boulardii*, and further research is needed to elucidate its mechanisms and long-term effects. Additionally, certain *Lactobacilli* and *Bifidobacteria* have been reported to reduce the pH in the intestinal lumen by producing lactic and acetic acids, thereby decreasing the production of IgA in mice [160, 161].

These results all suggest that probiotics play a significant role in maintaining the balance of gut microbial structure and could become a low-risk treatment option for IgAN. However, although probiotic supplements have demonstrated effectiveness under various conditions, using live strains can potentially lead to adverse events, particularly in children and adults with underlying health conditions. The primary concerns include live bacteria potentially causing bacteremia, the horizontal transfer of antibiotic resistance genes, excessive immune stimulation, and the formation of persistent colonies in susceptible individuals [162, 163]. To mitigate these risks, it is recommended that probiotic strains undergo rigorous evaluation before being marketed. Moreover, in clinical practice, alternatives such as prebiotics and heat-killed probiotics should be considered. These alternatives can offer the benefits of probiotics in certain scenarios without the associated risks posed by live bacteria.

### Hydroxychloroquine

Hydroxychloroquine (HCQ), a traditional antimalarial medication, is widely used for its anti-inflammatory and immunomodulatory effects in managing autoimmune conditions such as systemic lupus erythematosus and rheumatoid arthritis [164]. Specifically, HCQ exerts its immunoregulatory and anti-inflammatory effects by suppressing the signaling of TLRs in mucosal and renal tissues, suppressing the production of cytokines and chemokines, and inhibiting antigen presentation [165]. Given that proteinuria is the strongest prognostic factor in IgAN, its effect is dose-dependent and independent of other risk factors [166]. IgAN patients with proteinuria exceeding 1 g/d, or even 0.5 to 1 g/d, are considered to be at a high risk of kidney function decline [167]. Therefore, it is reasonable to believe that HCQ may reduce renal inflammation in IgAN patients by inhibiting TLR signaling in mucosal and renal tissues, modulating immune responses, increasing intracellular pH, stimulating nitric oxide synthesis, and reducing the production of Gd-IgA1. Consequently, these mechanisms effectively lower proteinuria.

A meta-analysis conducted by Zhang et al. [168] systematically searched PubMed and Embase databases, including five studies involving 587 participants, aiming to compare the effects of HCQ with other treatments in reducing proteinuria in patients with IgAN. The results indicated that although HCQ reduced proteinuria levels within six months, it did not show a significant advantage in the percentage reduction of proteinuria. The long-term effects of HCQ might be inferior to those of HCQ combined with renin-angiotensin-aldosterone system inhibitors (RAASi). The inclusion of both randomized controlled trials and retrospective studies in this meta-analysis might have impacted the consistency of the results due to design differences. Additionally, variations in HCQ dosage, treatment regimens, and study duration among the included studies could lead to reduced comparability of the results.

In a double-blind, randomized, placebo-controlled phase 2 clinical trial conducted by Liu et al. [169], a total of 60 patients undergoing RAASi treatment were enrolled and randomly assigned to either the HCQ group or the placebo group. The results showed a significant reduction in proteinuria levels in the HCQ group within 6 months (-48.4%), while the placebo group did not exhibit a significant change (10%). Additionally, the median proteinuria levels in the HCQ group were significantly lower than those in the placebo group. Furthermore, the baseline characteristics of the patients in both the HCQ and placebo groups were well-matched, ensuring high-quality evidence. However, due to the short duration of the study (6 months), the long-term efficacy and safety of HCQ could not be fully assessed. Based on these results to evaluate the long-term efficacy and safety of HCQ in IgAN patients, Liu et al. [170] subsequently conducted a single-center retrospective study involving 180 IgAN patients who had received at least one year of HCQ treatment. They analyzed changes in proteinuria and eGFR. The results showed a significant reduction in proteinuria levels at 12 months and 24 months, with decreases of 37.58% and 55.30%, respectively. The eGFR remained stable over the first 12 months but showed a decline at 24 months. These studies indicate that HCQ can significantly reduce proteinuria over a period of 6 to 24 months, suggesting its potential as an effective long-term therapeutic approach for managing IgAN.

Clinically, HCQ has gradually been incorporated into the treatment regimen for IgAN by some physicians, especially for patients who do not respond well to conventional treatments. Although no serious adverse events were reported, some patients experienced mild adverse effects such as dizziness, rashes, skin pigmentation, and gastrointestinal discomfort. These adverse effects might affect patient compliance, necessitating close monitoring in clinical practice. Additionally, current clinical studies on HCQ and IgAN are predominantly small-scale, single-center randomized controlled trials focusing on Chinese patients, lacking representation from different races and regions. Therefore, future research should include larger, multicenter randomized controlled trials with diverse populations to further validate the long-term efficacy and safety of HCQ.

### Fecal microbiota transplantation

FMT involves transferring a functional microbial community from the fecal matter of healthy donors into the gastrointestinal tract of recipients with microbial dysbacteriosis, aiming to re-establish a new gut microbiome. FMT is considered the best method for treating patients with *Clostridium difficile* infection and has been widely recognized and applied in clinical practice [171]. Its use in other diseases is still under research and clinical trials.

Barba et al. [172], investigating the impact of FMT on CKD mice induced by a 0.25% adenine diet for 4 weeks, discovered that FMT significantly improved the gut microbiome diversity in CKD mice, corrected gut microbial disarray, and reduced the release of uremic toxins via the intestinal cresol pathway. However, FMT did not significantly improve kidney function, serum creatinine levels, or inflammatory markers (such as TGFβ1, IL-6, TNFα) in CKD mice. This might be because CKD-induced kidney damage is not solely due to uremic toxins but also involves other factors such as systemic metabolic disorders and vascular lesions. These factors may persist even after toxin levels are reduced, leading to continued kidney damage. Therefore, merely improving gut microbiome might not be sufficient to significantly affect the systemic inflammatory response in CKD. Additionally, chronic structural damage in the kidneys (such as glomerulosclerosis and fibrosis) requires time to recover, and short-term reduction in toxin levels may not be enough to reverse the established tissue damage. A comprehensive approach addressing multiple pathological mechanisms of CKD is needed.

Currently, FMT has also emerged as a novel treatment approach for IgAN. Lauriero et al. [173] conducted a study that divided participants into healthy controls (HC), non-progressive IgAN patients (NP), progressive IgAN patients (P), and untreated groups. They first applied FMT by transplanting gut microbiome from different donors into α1KI-CD89Tg mice that had been treated with antibiotics via gavage. The results showed that FMT from the HC group significantly reduced albuminuria and the expression of the chemokine KC (CXCL1) in the kidneys of the mice and affected serum BAFF levels. This suggests that transplanting healthy microbiota might slow the progression of IgAN.

Research on the application of FMT in IgAN patients is still very limited, but some existing studies show potential for this treatment. One case report [174] indicated that two Chinese female patients with refractory IgAN received high-intensity fresh FMT via endoscopic transendoscopic enteral tubing for 6–7 months, followed by a 6-month follow-up. Both patients experienced a significant decrease in urinary protein excretion and an increase in serum albumin following enhanced FMT treatment, with their gut microbiome stabilizing, a decrease in *Proteobacteria*, and an increase in *Prevotella*. Regarding safety, patient A developed skin erythema and influenza during treatment, while patient B experienced transient diarrhea and discomfort related to endoscopic procedures, but no severe adverse events occurred.

Another case report [175] described a Chinese male patient with recurrent hematuria and proteinuria diagnosed with IgAN, who was treated with FMT capsules three times a month for one course and followed up for three months. The results showed that FMT capsule treatment significantly reduced urinary protein, eventually turning negative, and significantly increased gut microbiome diversity. The patient did not experience any significant adverse effects, but other clinical parameters were not reported in detail. Given that these case reports are based on only three patients, the generalizability of the results is limited. However, they preliminarily suggest that FMT might have potential in treating refractory IgAN. More large-scale, long-term randomized controlled trials are needed to confirm its long-term efficacy and safety. Therefore, further exploration of the therapeutic efficacy of FMT for IgAN is highly warranted.

### Glucocorticoids

For the past fifty years, glucocorticoids (GCs) have been one of the most effective and widely used medications for treating IgAN and preventing transplant rejection. They are particularly crucial in managing nephrotic syndrome and rapidly progressive glomerulonephritis caused by IgAN. GCs come in various derivatives with distinct biological properties and can be classified based on their duration of action into short-acting (8–12 h), intermediate-acting (12–36 h), and long-acting (36–72 h) categories. Despite the introduction of numerous novel immunosuppressants in recent years, which have significantly improved the treatment outcomes for IgAN, the essential role of GCs remains irreplaceable. A 2015 analysis of 32 single-center randomized controlled trials from the Cochrane database, involving 1781 patients, showed that GCs significantly improved the risk of needing a kidney transplant and doubling of serum creatinine while also effectively reducing proteinuria [176]. However, the benefits, optimal dosages, and lesser adverse effects of GCs treatment in patients with poor renal function—characterized by a glomerular filtration rate less than 60 ml/min/1.73 m², advanced CKD (eGFR < 30 ml/min/1.73 m²), mild to moderate proteinuria (0.5–1 g/24 h), and different pathological types of IgAN—remain controversial. Additionally, the reliability of this study is limited due to the prevalent high risk of bias, a lack of data identifying treatment-related harms, and generally short follow-up periods that do not fully assess long-term safety. Subsequently, a study again conducted a comprehensive analysis of the efficacy and safety of immunosuppressive therapy for IgAN based on the Cochrane database [177]. This multi-center randomized controlled study included 3,933 randomized participants, particularly targeting those with daily proteinuria exceeding 1 gram for treatment with GCs. The results indicated that short-term (typically two to four months) corticosteroid treatment, followed by gradual tapering, potentially benefits in preventing disease progression [177]. This study generally had longer follow-up periods, providing more extended observational data. However, during GCs treatment, many patients experienced severe adverse reactions such as increased infections and gastrointestinal discomfort, which could significantly impact treatment adherence and lead to the early termination of the study. Despite these issues, both studies were randomized controlled trials, and the latter’s multi-center design and larger sample size enhanced the reliability of the conclusions. The existing results support the efficacy of corticosteroids in reducing proteinuria in IgAN patients, but the risks of adverse events need to be carefully weighed. Clinical use should be cautious, with enhanced patient monitoring.

Beyond their therapeutic effects, an increasing number of adverse reactions have been reported during the treatment of IgAN with GCs. Given that GCs are secreted by the adrenal cortex and follow significant circadian rhythms—peaking in the early morning and reaching their lowest at night—the use of exogenous GCs, especially long-term or in high doses, can disrupt these intrinsic cortisol rhythms. This disruption may lead to an irregular distribution of hormone levels throughout the day, thereby affecting the body’s biological clock and related physiological processes [178]. Thaiss et al. [179], using 16 S rDNA sequencing and metagenomic sequencing, collected fecal samples every 6 h covering two day-night cycles to analyze the circadian rhythmic changes of the gut microbiome in mice and humans. The results demonstrated that the gut microbiome exhibit circadian rhythmic activities, closely related to the host’s diet, activity cycles, and hormone levels. When GCs interfere with the host’s circadian rhythms, the rhythms of the gut microbiome may also be affected. For example, dexamethasone can alter the diurnal fluctuation patterns of the gut microbiome in mice, particularly affecting the production of fatty acids and other metabolic products [180]. Wu et al. [181] further corroborated this by using male Wistar rats, divided into control and two different dosage groups of dexamethasone (DEX), finding that after 7 weeks of oral administration of dexamethasone sodium phosphate (DEX), rats showed slowed weight gain, increased fat tissue accumulation, dysregulation of lipid synthesis gene expression in the liver and adipose tissue, and significant reductions in gut microbiome abundance, diversity, and mucus secretion. These changes, which induce inflammatory cell infiltration, are closely related to disruptions in circadian rhythms and regulation of glucocorticoid receptor expression. Interestingly, the high-dose DEX group (0.05 mg/kg body weight per day) did not lead to fat accumulation in the liver; instead, it reduced liver fat content, which contradicts the general notion that GCs promote liver fat accumulation [182]. Similarly, in rodent models treated with GCs, specific gut microbiome such as *Firmicutes* and *Bacteroidetes* show altered proportions; specifically, an increase in the relative abundance of *Firmicutes* and a decrease in *Bacteroidetes*, leading to dysbacteriosis of the gut microbiome [183]. Mucin2 (MUC2), a mucin primarily expressed by colonic goblet cells, plays a crucial role in forming the intestinal mucus layer. This layer not only defends against endogenous and exogenous irritants and microbial adhesion and invasion but also allows the passage of nutrients, which is vital for maintaining intestinal barrier function and regulating interactions with the gut microbiome [184, 185]. GCs can weaken the protective function of the intestinal mucus layer by inhibiting the expression of MUC2, thus increasing the risk of pathogens and harmful substances penetrating the intestinal barrier [186]. However, in GF mice treated with DEX, mucin expression did not significantly differ from that of the control group, suggesting that the gut microbiome might be involved in the regulation of MUC2 expression by dexamethasone [186]. Another observational study [187], utilizing a polysaccharide-based testing method, collected urine samples at different time intervals (0–2 h, 2–5 h, 5–24 h) after subjects fasted overnight and ingested a solution containing various sugars, and quantitatively analyzed the sugar concentration in the urine using isocratic ion-exchange high-performance liquid chromatography. The results found that intestinal permeability in patients with IgAN was generally higher than in healthy individuals, although this increase was not specific to IgAN and occurred in various glomerular diseases. Long-term treatment with GCs in IgA patients may impact intestinal permeability, but these effects depend on the disease background and local treatment conditions [188]. Overall, GCs impact the function of the intestinal barrier to some extent, with effects likely related to disturbances in circadian rhythms and immunoregulatory mechanisms of the intestinal barrier. The specific mechanisms by which they affect changes in the gut microbiome require further investigation. Additionally, the gut microbiome also participates in the metabolic processes of GCs.

Nefecon, a new pharmaceutical containing budesonide, is formulated in a pH-sensitive capsule designed specifically for treating gut mucosal immunity in patients with IgAN. The drug is released at a specific location in the terminal ileum, near the ileocecal junction, an area rich in PPs and a primary site for the synthesis of Gd-IgA1. Here, Nefecon acts directly on the immune cells responsible for mucosal IgA production [189]. Due to its targeted release in the gut and high hepatic clearance rate, Nefecon has lower systemic exposure, thus reducing the common adverse effects associated with traditional GCs treatments.

The NEFIGAN trial (NCT01738035) is a randomized, double-blind, placebo-controlled Phase 2b study that recruited a total of 150 patients [190]. The trial comprised a 6-month run-in period, a 9-month treatment period, and a 3-month follow-up period. All participants received optimized renin-angiotensin system (RAS) blockade therapy as the baseline treatment, aiming to maximally suppress the RAS, reduce proteinuria, control hypertension, and protect renal function. The NEFIGAN trial assessed the additional therapeutic effects of Nefecon on top of this regimen. During the run-in period, the doses of ACEIs or ARBs were gradually increased to control the patients’ blood pressure to a target of less than 130/80 mm Hg and to reduce the urine protein-creatinine ratio (UPCR) to less than 0.5 g/g, or the 24-hour urine protein to less than 0.75 g/day. The results showed that after 9 months of treatment, Nefecon significantly reduced the UPCR, with reductions of 27.3% in the 16 mg/day group and 21.5% in the 8 mg/day group. The main adverse effects reported in the Nefecon group included deep vein thrombosis (occurring in the 16 mg/day Nefecon group) and unexplained deterioration of renal function during the follow-up period in patients whose dosage was reduced from 16 mg/day to 8 mg/day. This indicates that a higher dose (16 mg/day) of Nefecon is associated with a higher incidence of severe adverse effects. However, the main limitations of the NEFIGAN trial include the small sample size, the homogeneity of the patient population, and the lack of recent biopsy data for some patients. Additionally, due to differences in baseline treatments, the relative treatment effects may reflect the combined effects of both baseline and primary treatments, rather than the effects of Nefecon alone. These differences may affect the validity of the conclusions and are significant for interpreting the efficacy of Nefecon in the treatment of IgAN and its performance in various clinical trials.

In the Phase 3 NefigArd trial (NCT03643965), high-dose (16 mg/day) Nefecon treatment significantly reduced both the UPCR and the urine albumin-creatinine ratio (UACR) over a 9-month period compared to the placebo group, with an average UPCR reduction of 27%, consistent with previous studies, and maintained a relatively stable eGFR [191]. It is important to note that the proportion of patients with diabetes and prediabetes was higher in the Nefecon group at baseline, which might have influenced the results for proteinuria and eGFR. Additionally, at the 3-month mark, the Nefecon group showed a sharp increase in eGFR, a phenomenon that has not been fully explained and may be related to the drug’s hemodynamic effects or muscle wasting effects. Moreover, the safety of Nefecon was confirmed, with most adverse events being mild to moderate and reversible. Compared to other corticosteroid therapies, Nefecon did not lead to serious infections or long-term severe adverse effects [190, 191]. The most recent comprehensive clinical trial (NCT03643965) validated these earlier results, and the Nefecon group showed significantly less eGFR reduction compared to the placebo group over two years, with a 40.9% reduction in proteinuria [192]. Notably, adverse events were more frequent in the Nefecon-treated group compared to placebo, including but not limited to peripheral edema (17% vs. 4%), hypertension (12% vs. 3%), muscle spasms (12% vs. 4%), acne (11% vs. 1%), and headaches (10% vs. 8%) [192]. Despite some adverse reactions, the overall safety profile of Nefecon is acceptable, with no treatment-related deaths reported. Overall, Nefecon has demonstrated promising therapeutic effects and good tolerability and safety in treating IgAN. These findings support the broader clinical application of Nefecon in the future.

### IL-17 inhibitor

IL-17 inhibitors are a class of biologic agents used to treat autoimmune diseases by specifically targeting the IL-17 signaling pathway. These inhibitors effectively reduce the production of inflammatory mediators and the recruitment of inflammatory cells, thereby controlling inflammation driven by overactive Th17 cells. Despite increasing research indicating that IL-17 and Th17 cells play a central role in IgAN and other immune-mediated glomerular diseases, particularly through mechanisms related to the gut microbiome environment [82, 193, 194], the inhibition of IL-17 A has not consistently produced positive outcomes in some conditions, such as Crohn’s disease and IgAN treatment. Moreover, its effectiveness has shown limitations in certain renal disease models [195–197].

Uriol-Rivera et al. [198] first implemented an innovative combination therapy in seven patients with refractory IgAN, initiating treatment with the anti-inflammatory agent paricalcitol followed by IL-17 A blockade using secukinumab. Paricalcitol is a selective vitamin D receptor agonist that inhibits the differentiation of Th1 and Th17 cells and promotes the generation of Tregs, thereby suppressing excessive immune responses and inflammation. Paricalcitol is widely used in renal diseases primarily because it can reduce proteinuria, inhibit renal fibrosis, and protect renal function. Therefore, this combined therapy approach is rational, using paricalcitol first to modulate the immune system, providing a more stable immune environment for subsequent IL-17 inhibition. Their results demonstrated that the combination therapy significantly reduced proteinuria, with 71% of patients showing a decrease in proteinuria compared to baseline (eGFR < 60 ml/min/1.73 m²), and the annual decline rate of eGFR in patients decreased from 6.0 mL/min/1.73 m² before treatment to 2.3 mL/min/1.73 m² after treatment. By the end of the follow-up, occurrences of shingles, COVID-19, and mild to moderate candida infections were observed but were generally controllable. Interestingly, there was an increase in Th17.1 cell counts, which may reflect their crucial role in the pathology of refractory IgAN. However, as this was a single-center study with a limited sample size and lack of a control group, the reliability of the results is limited. Further large-scale studies are required to validate these findings.

### BAFF-APRIL inhibitor and anti-APRIL antibody

BAFF and APRIL are two critical cytokines in the immune system that play essential roles in the survival and function of B cells. In IgAN, BAFF and APRIL may exacerbate the formation of immune complexes and glomerular deposition by promoting B cell maturation and IgA production, particularly influencing the abnormal glycosylation of IgA1, thus playing a crucial role in the pathogenesis of IgAN [78]. Atacicept is a recombinant fusion protein composed of the Fc region of human IgG1 with the transmembrane activator and CAML interactor (TACI). By binding and inhibiting both the soluble and membrane-bound forms of BAFF and APRIL, Atacicept effectively reduces B cell numbers and interferes with their maturation, differentiation, and function, thereby modulating B cell-related immune responses [199].

The randomized, double-blind, placebo-controlled Phase 2a JANUS study [200] (NCT02808429), conducted in 2022, randomly assigned 16 patients into three groups: the atacicept 25 mg group (6 patients), the atacicept 75 mg group (5 patients), and the placebo group (5 patients). This study aimed to evaluate the safety, pharmacokinetic effects, and efficacy of atacicept in patients with IgAN. The results showed that atacicept demonstrated good safety, with most adverse events being mild to moderate (such as injection site reactions and urinary tract infections), and a few patients experiencing serious adverse events (such as acute kidney injury and viral gastroenteritis), which were not directly related to the treatment. Additionally, atacicept significantly reduced the levels of IgA, IgG, IgM, and Gd-IgA1 in the serum, and also led to a clinically significant reduction in 24-hour proteinuria while contributing to the stabilization of renal function. Notably, in the 25 mg dosage group, the improvement in proteinuria persisted until week 72. In contrast, although the 75 mg dosage group showed a greater reduction in Gd-IgA1, the improvement in proteinuria did not persist until week 72, Specifically, at week 48, three patients in the 75 mg dosage group experienced an increase in proteinuria, but by week 72, two of these patients had shown a decrease in proteinuria levels [200].

Recently, in the Phase 2b ORIGIN study (NCT04716231) [201], a total of 116 participants were recruited to evaluate the efficacy and safety of three doses of atacicept (25, 75, and 150 mg). At the 24-week and 36-week assessment points, atacicept groups showed significant reductions in the UPCR. Notably, the 150 mg dose demonstrated a more rapid and greater reduction in proteinuria (-41%) compared to the 75 mg dose (-28%), and it was the only dose that achieved a significant difference from placebo at 24 weeks (-41% vs. + 10%). By week 36, both the 150 mg and 75 mg dose groups continued to show significant reductions in UPCR, but the 150 mg group remained more effective, with a 40% reduction in UPCR compared to a 34% reduction in the 75 mg group. Despite the significant improvements in UPCR reduction at both 24 and 36 weeks for both doses, the consistency and effectiveness were more pronounced in the 150 mg group. Additionally, the safety outcomes were similar between the 75 mg and 150 mg dosage groups during the treatment period, providing necessary evidence for proceeding with a pivotal Phase III study [201]. Therefore, based on the overall efficacy and safety data, selecting the 150 mg dose of atacicept for further evaluation is promising for validating its potential long-term renal protective effects in the treatment of IgAN.

Sibeprenlimab (VIS649) is a humanized monoclonal antibody that binds to and neutralizes APRIL. Currently, Phase 1 and Phase 2 studies of sibeprenlimab have been completed. In the randomized, double-blind, single ascending dose Phase 1 clinical trial (NCT03719443), although sibeprenlimab exhibited nonlinear pharmacokinetics, it effectively inhibited the levels of APRIL and serum IgA in healthy volunteers across all studied doses, demonstrating good tolerability and safety [202]. Given that the Phase 1 clinical trial utilized a single ascending dose design, it primarily evaluated the short-term safety, tolerability, and preliminary pharmacokinetic characteristics of VIS649 in healthy volunteers.

The recent randomized, double-blind, placebo-controlled Phase 2 ENVISION study (NCT04287985) demonstrated that, compared to placebo, Sibeprenlimab significantly suppressed levels of APRIL and Gd-IgA1 in serum during the treatment period, leading to a notable reduction in proteinuria and improvement in renal function, particularly at higher doses (4 mg/kg and 8 mg/kg) [203]. At the end of 12 months of treatment, patients in these two dosage groups exhibited a greater reduction in the logarithmically adjusted urine protein/creatinine ratio compared to placebo, and this reduction persisted for 5 months post-treatment (16 months in total). Additionally, Sibeprenlimab demonstrated good short-term safety, with no increased risk of adverse events compared to the placebo group, although its long-term safety still needs further validation. Notably, during the 5-month follow-up period after treatment completion, patients in the Sibeprenlimab group showed a trend toward a return to baseline levels of serum IgA and Gd-IgA1, suggesting that continued suppression of APRIL may be necessary to maintain clinical efficacy [203].

### Dual blocker of angiotensin receptor and endothelial receptor

Sparsentan is a dual blocker of the angiotensin receptor and endothelin receptor. Angiotensin II (AngII) and Endothelin-1 (ET-1) are two key bioactive molecules in renal pathology, primarily produced by the renin-angiotensin system and endothelial cells. AngII is activated by angiotensin-converting enzyme and binds to the angiotensin II type 1 receptor in the kidneys, causing vasoconstriction, increasing glomerular pressure, and activating immune effector cells such as Th17 and Treg. This promotes inflammatory responses and fibrosis [204]. Concurrently, ET-1 binds to endothelin receptor A and B on podocytes and mesangial cells, causing vasoconstriction and mesangial cell proliferation, leading to a reduction in glomerular filtration rate and glomerulosclerosis [205]. These factors collectively contribute to the deposition of under-galactosylated IgA-antibody immune complexes in IgAN, stimulating cellular proliferation, exacerbating proteinuria and renal function decline, and leading to renal interstitial fibrosis [206, 207].

In a mouse model of IgAN (gddY), sparsentan slowed the progression of acute kidney injury and glomerulosclerosis, demonstrated a faster anti-proteinuric effect, and significantly protected podocytes and the endothelial glycocalyx, though it had no significant impact on circulating IgA levels [208]. Additionally, Reily et al. [209] evaluated the protective effects of sparsentan on IgAN by using engineered immune complexes (EIC) to induce glomerular injury in mice, simulating human IgAN. They found that sparsentan significantly reduced EIC-induced glomerular hyperplasia and abnormal expression of inflammatory genes at doses of 60 mg/kg and 120 mg/kg. Specifically, compared to the control group, sparsentan decreased the upregulation of complement genes, integrin components, MAPK family members, and Fc receptor elements. However, there may be conflicting views on the mechanism of action of sparsentan in different studies. The general consensus is that sparsentan works primarily by inhibiting ET-1 and RAS, while Reily et al. emphasized its role in regulating inflammatory gene expression. This could lead to different therapeutic strategies. For instance, if sparsentan acts mainly through ET-1 and RAS inhibition, combining ET-1 and RAS inhibitors might be more effective. Conversely, if its primary action is through modulating inflammatory gene expression, attention may need to be given to other anti-inflammatory agents.

Currently, sparsentan is undergoing a randomized, double-blind, Phase 3 PROTECT trial (NCT03762850) [210, 211]. Participants in this trial, despite having received at least 12 weeks of maximized renin-angiotensin system inhibition therapy, still exhibit proteinuria levels of 1.0 g/day or higher. In the 36-week assessment, the sparsentan group exhibited an average reduction of 49.8% in the urine protein-creatinine ratio, compared to a 15.1% reduction in the irbesartan group, and this reduction trend remained stable throughout the study [210]. In addition to changes in proteinuria, they also assessed changes in eGFR slope. The eGFR slope represents the rate of change in eGFR over time, indicating the kidney’s efficiency in filtering waste and excess fluid from the blood at specific times. At 110 weeks, the total 2-year slope of the sparsentan group, from the first day of treatment to week 110, was − 2.9 mL/min/1.73 m² per year, compared to -3.9 mL/min/1.73 m² per year for the irbesartan group. The chronic 2-year eGFR slope for the sparsentan group, from week 6 to week 110 of treatment, was − 2.7 mL/min/1.73 m² per year, while for the irbesartan group it was − 3.8 mL/min/1.73 m² per year [211]. These two slopes are designed to more comprehensively assess different aspects of the long-term impact of treatment on renal function. The chronic 2-year eGFR slope may exclude acute changes that could occur in the initial phase of treatment, thereby more accurately reflecting chronic efficacy or chronic changes in renal function. Although the incidence of adverse events in the sparsentan group was low, including dizziness, hypotension, and peripheral edema, and generally manageable, further studies are needed to understand the risks of long-term use [211]. Overall, these data preliminarily support sparsentan as a potential treatment option for patients with IgAN, particularly those with poor proteinuria control and ineffective treatment with existing RAS inhibitors. Therefore, future research should include longer-term follow-up to further confirm its long-term safety and efficacy. Additionally, the potential for its combination with other renoprotective drugs, such as SGLT2 inhibitors, should be explored.

## Conclusions

IgAN is the most common form of primary glomerulonephritis globally, with significant variability in incidence and severity across different populations, influenced by genetic, environmental, and dietary factors. We have comprehensively explored the complex role of mucosal immunity in the pathogenesis and management of IgAN, particularly the potential dominant role of gut mucosal immunity and the gut microbiome. Modulating the gut microbiome through specific therapeutic interventions can reduce the production of pathogenic IgA and alter immune responses, thereby preventing the formation of harmful immune complexes and potentially altering the course of the disease.

Current treatment strategies can be categorized into those related to the gut microbiome and emerging pharmacological approaches. Modulation of the gut microbiome through the use of antibiotics, probiotics, and fecal microbiota transplantation has shown potential benefits in adjusting the gut environment and reducing the recurrence rate of IgAN. Emerging therapeutic methods, including the use of BAFF/APRIL inhibitors, dual blockers of angiotensin receptor and endothelin receptor, and IL-17 inhibitors, have demonstrated significant potential in reducing inflammation and preserving renal function. The effectiveness and safety of these methods are being validated through clinical trials that have been approved by local institutional ethics committees and have obtained informed consent from patients and their families, ensuring confidentiality, fair participation, and the right to withdraw voluntarily. This offers potentially more effective and targeted treatment options for IgAN patients.

By clearly articulating the contributions of this research, we aim to provide clear directions for future studies. These studies should not only further explore the complex relationship between the gut microbiome and IgAN but also focus on developing and validating new therapeutic strategies to optimize and expand existing treatment options. Ultimately, this will improve the management of IgAN and reduce the global burden of this major glomerular disease. Future research should particularly focus on large-scale, multicenter clinical trials to more comprehensively validate and expand our findings, thereby providing more effective and personalized treatment options for IgAN patients.

## Data Availability

No datasets were generated or analysed during the current study.

## Abbreviations

IgAN: Primary Immunoglobulin A Nephropathy
C3: Complement component 3
IgM: Immunoglobulin M
IgG: Immunoglobulin G
MALT: Mucosa-associated lymphoid tissue
IBD: Inflammatory bowel disease
GALT: Gut-associated lymphoid tissue
PPs: Peyer’s patches
IECs: Intestinal epithelial cells
mIgA: Monomeric IgA
pIgA: Polymeric IgA
IgA: Immunoglobulin A
PCs: Plasma cells
LP: Lamina propria
SIgA: Secretory IgA
GF: Germ-free
SFB: Segmented filamentous bacteria
Tregs: Regulatory T cells
Gd-IgA1: Galactose-deficient IgA1
M cells: Microfold cells
TGF-β: Transforming growth factor-beta
C1GALT1: Core-1 β1,3-galactosyltransferase
Cosmc: Core-1 β3-Gal-T-specific molecular chaperone 1
BAFF: B cell activating factor
APRIL: A proliferation-inducing ligand
TLRs: Toll-like receptors
AAT: Alpha-1 antitrypsin
TLR4: Toll-like receptor 4
GWAS: Genome-wide association studies
tTGA: Tissue transglutaminase antibodies
LPS: Lipopolysaccharide
FMT: Fecal microbiota transplantation
CKD: Chronic kidney disease
PPIs: Proton pump inhibitors
CsA: Cyclosporine A
MMF: Mycophenolate mofetil
MPA: Mycophenolic acid
MPAG: Mycophenolic acid glucuronide
GUS: β-glucuronidase
FMN: Flavin mononucleotide
PICs: Programmable inhibitory cells
OPV: Oral poliovirus vaccine
HCQ: Hydroxychloroquine
RAASi: Renin-angiotensin-aldosterone system inhibitors
GCs: Glucocorticoids
DEX: Dexamethasone sodium phosphate
MUC2: Mucin2
RAS: Renin-angiotensin system
UPCR: Urine protein-creatinine ratio
UACR: Urine albumin-creatinine ratio
eGFR: Estimated glomerular filtration rate
AngII: Angiotensin II
ET-1: Endothelin-1
TACI: Transmembrane activator and CAML interactor
EIC: Engineered immune complexes
